# Supplementary material for: Exploring disaster impacts on adaptation actions in 549 cities worldwide
Source: Nat Commun. 2022 Jun 10;13:3360. doi: 10.1038/s41467-022-31059-z (PMC9187717; doi:10.1038/s41467-022-31059-z)
Supplement: Supplementary file 1 — Supplementary Information file [file 41467_2022_31059_MOESM1_ESM.pdf]

Supplementary Information for:

Exploring disaster impacts on adaptation actions in 549 cities worldwide

Daniel Nohrstedt[1,2,*], Jacob Hileman[1,2], Maurizio Mazzoleni[2,3,4], Giuliano Di Baldassarre[2,4], and Charles F. Parker[1,2]
[1]Department of Government, Uppsala University, Sweden; [2]Centre of Natural Hazards and Disaster Science (CNDS), Uppsala University, Sweden; [3]Institute for Environmental Studies, Vrije Universiteit Amsterdam, the Netherlands; [4]Department of Earth Sciences, Uppsala University, Sweden.
*Corresponding author, email: daniel.nohrstedt@statsvet.uu.se

Contents

Section 1. Study sample ... 2
Section 2: Adaptation Action Coding Procedures ... 12
Coding Adaptation Actions and Natural Hazard Events by Geographic Region ... 12
Coding Adaptation Actions by Hazard Type ... 12
Inter-coder reliability test of adaptation action type categorization ... 23
Section 3: Dependent, Independent, and Control Variables ... 24
Variable descriptions ... 24
Adaptive Capacity Control Variables ... 25
Section 4: Full Regression Results for All Types of Adaptation Actions ... 28
Supplementary References ... 72

Supplementary Information summary

One of the core contributions of this study is to develop and apply a categorization of different adaptation actions that cities may take. To do so, we specifically focus on (a) the description of the action as submitted to the CDP, and (b) the relationship between the action and the presence (or absence) of different natural hazard event types. This is the basis for empirically exploring whether disaster frequency and severity have different impacts across adaptation action types. The article distinguishes between four adaptation action types: “specific disaster adaptation”, “expansive adaptation”, “generic preparedness” and “other” adaptation actions. In the first section of the Supplementary Information, we provide a map (Supplementary Figure A1) and detailed list (Supplementary Table A1) of all the locations included in the analysis. In the second section, we detail how we defined the coding scheme and implemented the coding process to differentiate all four types of adaptation action (Supplementary Figure B1), and provide the complete list of adaptation action descriptions contained in the CDP dataset (Supplementary Table B1). In the third section, we provide the full list and descriptions of all dependent, independent, and control variables (Table C1). In the final section, we include the full set of expanded regression results for all types of adaptation action (Tables D1-D17).

Section 1. Study sample

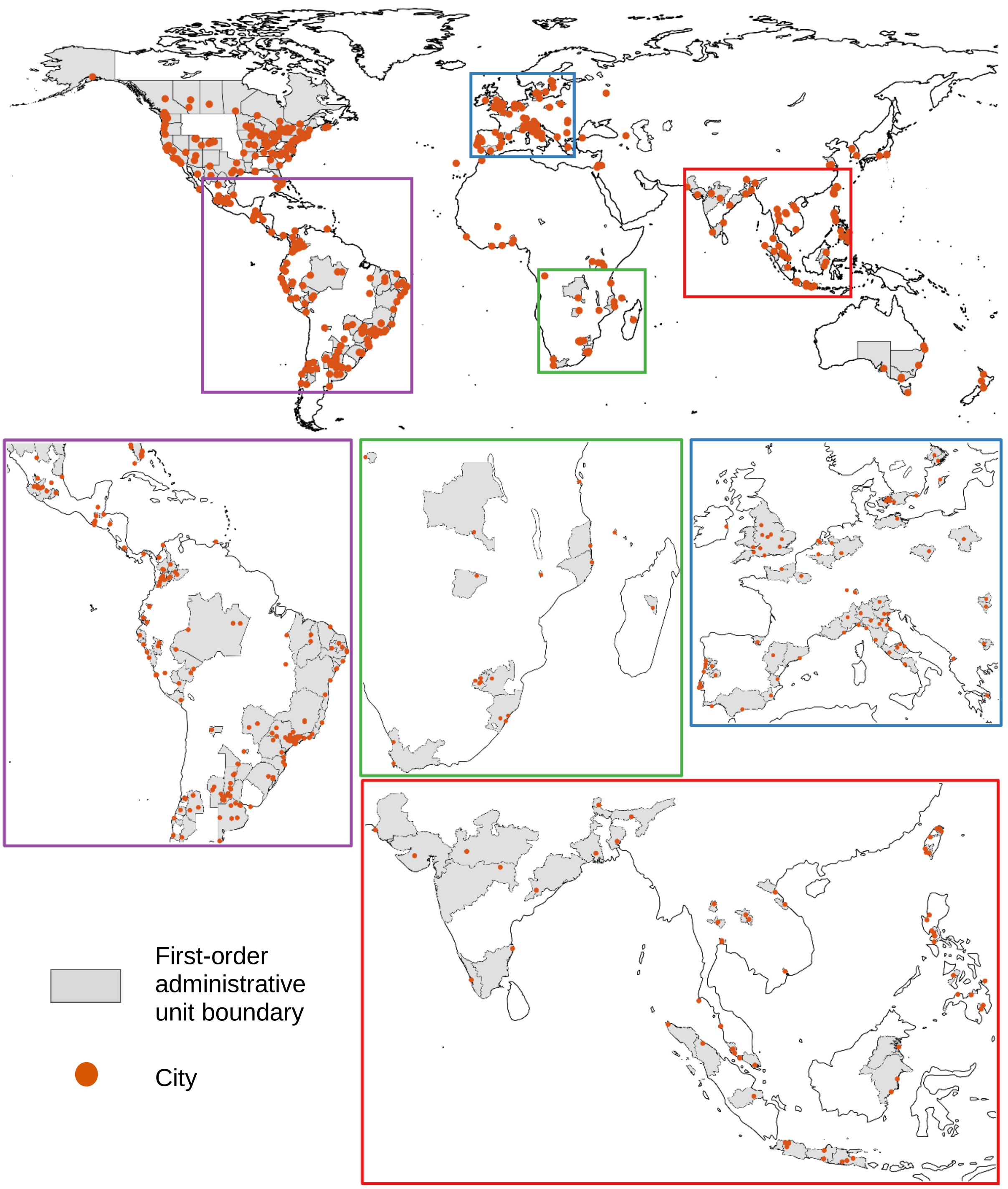

Supplementary Figure 1. Locations of cities by province (first-order administrative unit). Cities listed in Supplementary Table A1. Locations determined using data from World Cities Database (Basic version, updated July 2021, downloaded February 23, 2022 from: https://simplemaps.com/data/world-cities), and GeoNames (downloaded February 23, 2022 from: https://public.opendatasoft.com/explore/dataset/geonames-all-cities-with-a-population-1000/information/?disjunctive.cou_name_en&sort=name&location=12,45.72056,9.82229&basemap=jawg.light). Maps created in Matlab R2021b.

Supplementary Table 1: List of all 549 cities included in the analysis

Country/ State	Income Group	City	City Pop.
Albania	Upper middle	Tirana	780531
Argentina	Upper middle	Arias	3022914
Argentina	Upper middle	Arteaga	30446
Argentina	Upper middle	Avellaneda	31294
Argentina	Upper middle	Bell Ville	NA
Argentina	Upper middle	Buenos Aires	50546
Argentina	Upper middle	Camilo Aldao	111708
Argentina	Upper middle	Carmen de Patagones	15520
Argentina	Upper middle	Caseros	8714
Argentina	Upper middle	Chacabuco	24111
Argentina	Upper middle	Chañar Ladeado	NA
Argentina	Upper middle	Córdoba	270929
Argentina	Upper middle	Correa	299123
Argentina	Upper middle	Corrientes	7249
Argentina	Upper middle	General Alvear	35281
Argentina	Upper middle	Godoy Cruz	5209
Argentina	Upper middle	Malabrigo	1430000
Argentina	Upper middle	Malargue	6285
Argentina	Upper middle	Mendoza	8257
Argentina	Upper middle	Monte Buey	376528
Argentina	Upper middle	Olavarría	6221
Argentina	Upper middle	Oro Verde	247863
Argentina	Upper middle	Paraná	46429
Argentina	Upper middle	Pérez	191903
Argentina	Upper middle	Rauch	27660
Argentina	Upper middle	Reconquista	115041
Argentina	Upper middle	Resistencia	29472
Argentina	Upper middle	Rosario	3546
Argentina	Upper middle	Salliqueló	5415
Argentina	Upper middle	San Antonio de Areco	6121
Argentina	Upper middle	San Isidro	7339
Argentina	Upper middle	San Justo	29204
Argentina	Upper middle	San Martín de los Andes	78570
Argentina	Upper middle	Santa Fé Ciudad	982137
Argentina	Upper middle	Soldini	23943
Argentina	Upper middle	Totoras	398927
Argentina	Upper middle	Venado Tuerto	3212
Argentina	Upper middle	Vicente López	10292
Argentina	Upper middle	Villa General Belgrano	76432
Australia	High	Adelaide	33571
Australia	High	Byron Shire	236724
Australia	High	Hobart	25709
Australia	High	Melbourne	52062
Australia	High	Rosebud	164436
Australia	High	Sydney	160862
Bangladesh	Lower middle	Dhaka	5100000
Belgium	High	Brussels	179277
Bolivia	Lower middle	Tarija	254211
Brazil	Upper middle	Águas da Prata	80291
Brazil	Upper middle	Angra dos Reis	18411
Brazil	Upper middle	Aracaju	1021127
Brazil	Upper middle	Araçatuba	86839
Brazil	Upper middle	Assis	2057711
Brazil	Upper middle	Bertioga	10852
Brazil	Upper middle	Betim	2857329
Brazil	Upper middle	Boa Ventura	2635365
Brazil	Upper middle	Bonito	360216
Brazil	Upper middle	Botucatu	11258
Brazil	Upper middle	Brumadinho	21487
Brazil	Upper middle	Brusque	786797
Brazil	Upper middle	Campina Grande	378089
Brazil	Upper middle	Campinas	33973
Brazil	Upper middle	Campo Grande	34909
Brazil	Upper middle	Canoas	410332

Brazil	Upper middle	Capivari	5751
Brazil	Upper middle	Carnaúba dos Dantas	1751907
Brazil	Upper middle	Cioeste	506701
Brazil	Upper middle	Cordeirópolis	291855
Brazil	Upper middle	Cruzeiro do Sul	112463
Brazil	Upper middle	Cubatão	1635766
Brazil	Upper middle	Curitiba	797029
Brazil	Upper middle	Epitaciolândia	169511
Brazil	Upper middle	Extrema	503812
Brazil	Upper middle	Florianópolis	182082
Brazil	Upper middle	Fortaleza	6520266
Brazil	Upper middle	Franco da Rocha	7429
Brazil	Upper middle	Guarujá	341343
Brazil	Upper middle	Igarassu	1444226
Brazil	Upper middle	Itacoatiara	230813
Brazil	Upper middle	Itatiba	71554
Brazil	Upper middle	Joinville	128818
Brazil	Upper middle	Londrina	508750
Brazil	Upper middle	Lorena	590466
Brazil	Upper middle	Maceió	8137
Brazil	Upper middle	Mairiporã	195374
Brazil	Upper middle	Manaus	103000
Brazil	Upper middle	Niterói	60516
Brazil	Upper middle	Nova Friburgo	144000
Brazil	Upper middle	Palmas	1189234
Brazil	Upper middle	Pau Brasil	48576
Brazil	Upper middle	Porto Alegre	2500000
Brazil	Upper middle	Presidente Prudente	25000
Brazil	Upper middle	Recife	118720
Brazil	Upper middle	Rio de Janeiro	131302
Brazil	Upper middle	Salvador	290752
Brazil	Upper middle	Santo André	115051
Brazil	Upper middle	Santos	88098
Brazil	Upper middle	São Bento do Sapucaí	98374
Brazil	Upper middle	São Cristóvão	225635
Brazil	Upper middle	São João da Boa Vista	676407
Brazil	Upper middle	São José dos Campos	434742
Brazil	Upper middle	São Leopoldo	11000
Brazil	Upper middle	São Paulo	90089
Brazil	Upper middle	São Vicente	708581
Brazil	Upper middle	Sorocaba	11253503
Brazil	Upper middle	Sumaré	333000
Brazil	Upper middle	Tatuí	661833
Brazil	Upper middle	Teresina	237007
Brazil	Upper middle	Tremembé	116296
Brazil	Upper middle	Venâncio Aires	40984
Brazil	Upper middle	Vila Nova dos Martírios	641523
Brazil	Upper middle	Vitória	78864
Bulgaria	Upper middle	Sofia	1402666
Burkina Faso	Low	Bakata	39308
Canada	High	Ajax	1256788
Canada	High	Bridgewater	978421
Canada	High	Calgary	52898
Canada	High	Durham	74003
Canada	High	Edmonton	637329
Canada	High	Guelph	92000
Canada	High	Halifax	228400
Canada	High	Hamilton	718027
Canada	High	Kingston	8532
Canada	High	Kitchener	441400
Canada	High	Laval	119677
Canada	High	London, ON	673070
Canada	High	Mississauga	131794
Canada	High	Montréal	557541
Canada	High	North Vancouver	124000
Canada	High	Peterborough	242368
Canada	High	Prince George	385288
Canada	High	Saskatoon	721599
Canada	High	St Catharines	82094
Canada	High	Sudbury	131400
Canada	High	Thunder Bay	160765
Canada	High	Toronto	107909
Canada	High	Vancouver	2915721

Canada	High	Victoria	136235
Canada	High	Whitby	437413
Canada	High	Windsor	1942044
Canada	High	Winnipeg	270475
Chile	High	Cerro Navia	223574
Chile	High	Concepción	166080
Chile	High	Peñalolén	220357
Chile	High	Providencia	132622
Chile	High	Santiago	241599
Chile	High	Talca	141370
Chile	High	Valdivia	3508741
Chile	High	Vitacura	85000
China	Upper middle	Nanjing	8335000
China	Upper middle	Zhenjiang	3186300
Colombia	Upper middle	Armenia	42280
Colombia	Upper middle	Barrancabermeja	2514161
Colombia	Upper middle	Cartagena	136398
Colombia	Upper middle	Cartago	37255
Colombia	Upper middle	Ibagué	971592
Colombia	Upper middle	Leticia	3694
Colombia	Upper middle	Madrid (Colombia)	400136
Colombia	Upper middle	Manizales	152655
Colombia	Upper middle	Medellín	82118
Colombia	Upper middle	Palmira	27932
Colombia	Upper middle	Pereira	301226
Colombia	Upper middle	Rionegro	472000
Colombia	Upper middle	San Pedro de Urabá	191704
Colombia	Upper middle	Santiago de Cali	564077
Colombia	Upper middle	Sopó	133652
Colombia	Upper middle	Tópaga	308671
Colombia	Upper middle	Tulua	2369829
Colombia	Upper middle	Yopal	209086
Comoros	Lower middle	Moroni	52959
Congo (Democratic Republic)	Low	Lubumbashi	5652000
Congo (Democratic Republic)	Low	Ville de Kinshasa	17071000

Costa Rica	Upper middle	La Unión	108990
Costa Rica	Upper middle	San Jose, CR	342188
Denmark	High	Copenhagen	602481
Denmark	High	Egedal	42531
Denmark	High	Gladsaxe	68919
Denmark	High	Hillerød	50104
Denmark	High	Hvidovre	52672
Denmark	High	Hørsholm	24856
Denmark	High	Roskilde	87276
Ecuador	Upper middle	Cuenca	505585
Ecuador	Upper middle	Quito	2440553
Ecuador	Upper middle	Santiago de Guayaquil	2239191
France	High	Le Havre	2257268
France	High	Nice	172769
France	High	Paris	550000
Georgia	Upper middle	Tbilisi	1164850
Germany	High	Cologne	58043
Germany	High	Greifswald	1069192
Ghana	Lower middle	Accra	2230894
Greece	High	Athens	664046
Guatemala	Upper middle	Escuintla	272300
Guatemala	Upper middle	Guanagazapa	170543
Guatemala	Upper middle	Guatemala City	20083
Guatemala	Upper middle	Iztapa	11917
Guatemala	Upper middle	Puerto Barrios	56907
Guatemala	Upper middle	San Jose	992541
Guatemala	Upper middle	San Pedro Carchá	97000
Guatemala	Upper middle	Santa Catarina Pinula	1200000
Guatemala	Upper middle	Villanueva	116000
Honduras	Lower middle	Tegucigalpa	1225043
India	Lower middle	Chennai	1498970
India	Lower middle	Gangtok	1286995
India	Lower middle	Guwahati	633553
India	Lower middle	Indore	2197447
India	Lower middle	Jaipur	2405665
India	Lower middle	Kochi	3046163

India	Lower middle	Kolkata	100286
India	Lower middle	Nagpur	7720000
India	Lower middle	Rajkot	14961394
Indonesia	Lower middle	Balikpapan	259913
Indonesia	Lower middle	Banda Aceh	2139891
Indonesia	Lower middle	Bekasi	1729428
Indonesia	Lower middle	Blitar	422732
Indonesia	Lower middle	Bogor	140971
Indonesia	Lower middle	Bontang	861414
Indonesia	Lower middle	Jakarta	233123
Indonesia	Lower middle	Jambi	636012
Indonesia	Lower middle	Malang	170611
Indonesia	Lower middle	Medan	10075310
Indonesia	Lower middle	Probolinggo	591134
Indonesia	Lower middle	Semarang	253026
Indonesia	Lower middle	Tangerang City	2247425
Indonesia	Lower middle	Tarakan	2873484
Indonesia	Lower middle	Yogyakarta	1081009
Israel	High	Tel Aviv-Yafo	441375
Italy	High	Bologna	69439
Italy	High	Bolzano	119252
Italy	High	Ferrara	54586
Italy	High	Genova	983755
Italy	High	La Spezia	389984
Italy	High	L'Aquila	132053
Italy	High	Massa Marittima	196000
Italy	High	Milano	157663
Italy	High	Napoli	150381
Italy	High	Padova	2876614
Italy	High	Parma	580209
Italy	High	Pescara	93787
Italy	High	Ravenna	1395274
Italy	High	Rimini	881608
Italy	High	Roma	107669
Italy	High	Teramo	8303
Italy	High	Torino	211210
Italy	High	Venezia	261245
Ivory Coast	Lower middle	Cocody	800000

Japan	High	Nagoya	2303070
Japan	High	Tokyo	20536207
Japan	High	Yokohama	13857554
Jordan	Upper middle	Amman	3920000
Kenya	Lower middle	Kisumu	968909
Kenya	Lower middle	Nairobi	4500000
Kenya	Lower middle	Nakuru	2038945
Korea (the Republic of)	High	Changwon	1241635
Korea (the Republic of)	High	Seoul	1072657
Korea (the Republic of)	High	Suwon	10104552
Madagascar	Low	Antananarivo	1370326
Malawi	Low	Blantyre	997258
Malaysia	Upper middle	Iskandar	1976000
Malaysia	Upper middle	Jasin	141000
Malaysia	Upper middle	Melaka	524353
Malaysia	Upper middle	Petaling Jaya	1085000
Malaysia	Upper middle	Seberang Perai	658611
Malaysia	Upper middle	Sepang	207354
Malaysia	Upper middle	Shah Alam	669894
Mexico	Upper middle	Chapala	215178
Mexico	Upper middle	Chihuahua	858555
Mexico	Upper middle	Degollado	1391180
Mexico	Upper middle	Guadalajara	658414
Mexico	Upper middle	Hermosillo	601263
Mexico	Upper middle	JICOSUR	NA
Mexico	Upper middle	JIMAV	3730131
Mexico	Upper middle	Juárez	147918
Mexico	Upper middle	La Barca	371457
Mexico	Upper middle	La Paz	85000
Mexico	Upper middle	Mexico City	NA
Mexico	Upper middle	Morelia	664193
Mexico	Upper middle	NA	477117
Mexico	Upper middle	Naucalpan de Juárez	916796
Mexico	Upper middle	San Pedro Tlaquepaque	8833416

Mexico	Upper middle	Tampico	784776
Mexico	Upper middle	Tonalá	727267
Mexico	Upper middle	Torreón	314418
Mexico	Upper middle	Victoria, MX	346029
Morocco	Lower middle	Casablanca	3360000
Mozambique	Low	Nacala	211581
Mozambique	Low	Pemba	225182
Netherlands	High	Nijmegen	175000
Netherlands	High	Rotterdam	650737
Netherlands	High	The Hague	532392
New Zealand	High	Auckland	1637059
New Zealand	High	Hutt City	82765
New Zealand	High	New Plymouth District	104700
New Zealand	High	Wellington	212898
Nigeria	Lower middle	Ibadan	22910709
Nigeria	Lower middle	Lagos	5591589
Pakistan	Lower middle	Karachi	25000000
Palestine	Lower middle	Abasan Al-Kabira	31333
Palestine	Lower middle	Bani-Suhaila	42000
Palestine	Lower middle	Ramallah	66000
Panama	High	Chorrera	161470
Paraguay	Upper middle	Asunción	525252
Peru	Upper middle	Arequipa	338000
Peru	Upper middle	Ate	984189
Peru	Upper middle	Bellavista	74000
Peru	Upper middle	Callao	994494
Peru	Upper middle	Chiclayo	434659
Peru	Upper middle	Chimbote	971116
Peru	Upper middle	Comas	597963
Peru	Upper middle	Cusco	647000
Peru	Upper middle	Independencia	553000
Peru	Upper middle	Jesús María	91670
Peru	Upper middle	Lima	77955
Peru	Upper middle	Magdalena del Mar	9302869
Peru	Upper middle	Miraflores	60091

Peru	Upper middle	Piura	97298
Peru	Upper middle	San Borja	113540
Peru	Upper middle	Santiago de Surco	503044
Peru	Upper middle	Tahuamanu	2500
Peru	Upper middle	Tarapoto	484475
Peru	Upper middle	Trujillo	186300
Philippines	Lower middle	Baguio City	354170
Philippines	Lower middle	Batangas	353800
Philippines	Lower middle	Cagayan de Oro	20000
Philippines	Lower middle	Davao	252074
Philippines	Lower middle	Del Carmen	345366
Philippines	Lower middle	Dipolog City	1632991
Philippines	Lower middle	La Carlota	259444
Philippines	Lower middle	Malolos	504509
Philippines	Lower middle	Muntinlupa	3046335
Philippines	Lower middle	Quezon City	675950
Philippines	Lower middle	San Carlos City	64469
Philippines	Lower middle	Santa Rosa	132536
Philippines	Lower middle	Tagum City	130759
Poland	High	Warsaw	639600
Poland	High	Wroclaw	1763203
Portugal	High	Amarante	38598
Portugal	High	Cascais	55398
Portugal	High	Castelo Branco	52703
Portugal	High	Faro	62125
Portugal	High	Figueira da Foz	61033
Portugal	High	Funchal	208122
Portugal	High	Ílhavo	547733
Portugal	High	Lisbon	79465
Portugal	High	Ovar	111892
Portugal	High	Porto	56217
Portugal	High	Torres Vedras	237591
Portugal	High	Viseu	99274
Romania	Upper middle	Alba-Iulia	74000
Romania	Upper middle	Cluj-Napoca	309338
Russian Federation	Upper middle	Moscow	12615300
Sierra Leone	Low	Freetown	NA

South Africa	Upper middle	Cape Town	3379104
South Africa	Upper middle	Durban	4950000
South Africa	Upper middle	Ekurhuleni	383864
South Africa	Upper middle	Johannesburg	3306198
South Africa	Upper middle	KwaDukuza	21044548
South Africa	Upper middle	Mogale City	276719
South Africa	Upper middle	Pietermaritzburg	618536
South Africa	Upper middle	Pretoria - Tshwane	278749
South Africa	Upper middle	Steve Tshwete	4199038
South Africa	Upper middle	West Coast District Municipality	391766
Spain	High	Barcelona	572000
Spain	High	Málaga	665909
Spain	High	Murcia	251604
Spain	High	Valencia	1612765
Spain	High	Vitoria-Gasteiz	443243
Spain	High	Zaragoza	791143
Sweden	High	Karlskrona	66675
Sweden	High	Malmö	41708
Sweden	High	Stockholm	334760
Sweden	High	Uppsala	943700
Sweden	High	Visby	225164
Switzerland	High	Basel	199698
Switzerland	High	Zürich	428058
Taiwan, Greater China	High	Kaohsiung	2774498
Taiwan, Greater China	High	New Taipei	3986689
Taiwan, Greater China	High	Pingtung County	826104
Taiwan, Greater China	High	Taichung	2798029
Taiwan, Greater China	High	Tainan	1883831
Taiwan, Greater China	High	Taoyuan	2220872
Taiwan, Greater China	High	Yilan County	455683
Tanzania	Low	Dar es Salaam	6041000
Thailand	Upper middle	Bangkok	5678570
Thailand	Upper middle	Hat Siao	119265
Thailand	Upper middle	Khon Kaen	49841
Thailand	Upper middle	Mahasarakham	83045
Thailand	Upper middle	Nakhon Sawan	255167
Thailand	Upper middle	Nonthaburi	20029
Thailand	Upper middle	Patong	6761
Turkey	Upper middle	Istanbul	15067724
Uganda	Low	Kampala	1631210
United Kingdom	High	Bath	188600
United Kingdom	High	Bournemouth	193700
United Kingdom	High	Cambridge	124919
United Kingdom	High	Cardiff	360100
United Kingdom	High	Coventry	351520
United Kingdom	High	Leicester	8883800
United Kingdom	High	London	2805685
United Kingdom	High	Manchester	153866
United Kingdom	High	Somerset West and Taunton	262470
United Kingdom	High	Wolverhampton	365000
United States of America	High	Abington	296424
United States of America	High	Alameda	72827
United States of America	High	Anchorage	1637645
United States of America	High	Ann Arbor	88468
United States of America	High	Arlington	75961
United States of America	High	Asheville	27977
United States of America	High	Ashland	121643
United States of America	High	Aspen	4603
United States of America	High	Atlanta	265070
United States of America	High	Aurora	39627
United States of America	High	Austin	60439
United States of America	High	Baltimore	63969
United States of America	High	Beaverton	11918
United States of America	High	Bellingham	63157
United States of America	High	Benicia	234962
United States of America	High	Berkeley	527438

United States of America	High	Beverly	160507
United States of America	High	Bloomington	202498
United States of America	High	Boston	470130
United States of America	High	Boulder	4021488
United States of America	High	Boynton Beach	35321
United States of America	High	Breckenridge	424452
United States of America	High	Brisbane, CA	66772
United States of America	High	Broward	11378
United States of America	High	Buffalo	110146
United States of America	High	Burlington	504730
United States of America	High	Carmel	877625
United States of America	High	Charlotte	1046079
United States of America	High	Charlottesville	90509
United States of America	High	Chicago	93532
United States of America	High	Chula Vista	64743
United States of America	High	Cincinnati	92478
United States of America	High	Cleveland	34399
United States of America	High	Coalville	9003
United States of America	High	Columbia, MO	108540
United States of America	High	Columbus	5035
United States of America	High	Culver City	701222
United States of America	High	Cupertino	5406
United States of America	High	Dallas	75374
United States of America	High	Dedham	1870000
United States of America	High	Denver	147500
United States of America	High	Detroit	25777
United States of America	High	Dublin, CA	36000
United States of America	High	Dubuque	470914
United States of America	High	Durham, NC	134037
United States of America	High	East Hampton, NY	56000
United States of America	High	Easton	12500
United States of America	High	Eau Claire	266000
United States of America	High	Emeryville	5811

United States of America	High	Encinitas	110533
United States of America	High	Eugene	450182
United States of America	High	Evanston	145674
United States of America	High	Fayetteville	992743
United States of America	High	Flagstaff	201522
United States of America	High	Fremont	2710006
United States of America	High	Fresno	75603
United States of America	High	Greenbelt	29767
United States of America	High	Greensboro	19501
United States of America	High	Gretna	84524
United States of America	High	Guilford	93510
United States of America	High	Hayward	867235
United States of America	High	Highland Park	102245
United States of America	High	Hillsborough	32053
United States of America	High	Hollywood	75344
United States of America	High	Honolulu	770645
United States of America	High	Houston	17935
United States of America	High	Huntington Beach	393292
United States of America	High	Indianapolis	66937
United States of America	High	Iowa City	608579
United States of America	High	Key West	23000
United States of America	High	Knoxville	17765
United States of America	High	La Crosse	55310
United States of America	High	Lake Forest	41648
United States of America	High	Lake Worth	674875
United States of America	High	Lakewood	25377
United States of America	High	Lancaster, PA	31394
United States of America	High	Las Vegas	95120
United States of America	High	Lexington, MA	28549
United States of America	High	Long Beach	43146
United States of America	High	Los Angeles	80648
United States of America	High	Louisville	227471
United States of America	High	Madison	673104

United States of America	High	Manhattan Beach	421538
United States of America	High	Medford	26928
United States of America	High	Memphis	118742
United States of America	High	Miami	883881
United States of America	High	Minneapolis	305580
United States of America	High	Miramar	650571
United States of America	High	Moab	31822
United States of America	High	Nashville	18000
United States of America	High	New Bedford	149923
United States of America	High	New Orleans	258612
United States of America	High	New York City	21457
United States of America	High	Norfolk	8580187
United States of America	High	Northampton	209556
United States of America	High	Oakland	28027
United States of America	High	Palo Alto	90048
United States of America	High	Park City	859035
United States of America	High	Philadelphia	267316
United States of America	High	Phoenix	292472
United States of America	High	Piedmont	142830
United States of America	High	Portland, ME	119045
United States of America	High	Portland, OR	243578
United States of America	High	Princeton	300467
United States of America	High	Providence	391121
United States of America	High	Richmond, CA	287208
United States of America	High	Richmond, VA	21117
United States of America	High	Rochester	97514
United States of America	High	Sacramento	165885
United States of America	High	Salem	57797
United States of America	High	Salt Lake City	717259
United States of America	High	San Antonio	26962
United States of America	High	San Francisco	60000
United States of America	High	San Jose, CA	1557505
United States of America	High	San Leandro	178948
United States of America	High	Santa Barbara	186239
United States of America	High	Santa Cruz, CA	651373
United States of America	High	Santa Fe	667560
United States of America	High	Santa Monica	1247633
United States of America	High	Sarasota	1345047
United States of America	High	Saratoga Springs	2337748
United States of America	High	Savannah	1469845
United States of America	High	Seattle	41106
United States of America	High	Secaucus	5288
United States of America	High	Snoqualmie	8243
United States of America	High	Somerville	193496
United States of America	High	South Bend	42284
United States of America	High	South Miami	2121
United States of America	High	St Louis	220742
United States of America	High	St. Petersburg	47514
United States of America	High	Surfside	246256
United States of America	High	Tacoma	226087
United States of America	High	Takoma Park	86570
United States of America	High	Toledo	154684
United States of America	High	Vail	727842
United States of America	High	Washington, DC	13516
United States of America	High	West Hollywood	214135
United States of America	High	West Palm Beach	698415
United States of America	High	Wilmington	67000
United States of America	High	Winona	51320
United States of America	High	Winston-Salem	258054
Uruguay	High	Montevideo	1380432
Venezuela	Upper middle	Maneiro	NA
Vietnam	Lower middle	Dong Hoi City	8611000
Vietnam	Lower middle	Ho Chi Minh	323800
Vietnam	Lower middle	Vinh City	130950
Zambia	Lower middle	Mazabuka	182205

Section 2: Adaptation Action Coding Procedures

Coding Adaptation Actions and Natural Hazard Events by Geographic Region

In order to ensure spatial proximity between the locations of the natural hazard events in the EM-DAT dataset and the adaptation actions in the CDP dataset, we reviewed the geographic information provided and determined the first-order administrative unit – the largest sub-national territorial unit within a country – where these events occurred. Given the CDP adaptation actions dataset provides both the country and city names, the geocoding process was a straightforward matching exercise using a global list of first-order administrative units[1]. The EM-DAT dataset provides information containing country names and a non-uniform, subjective description of the subnational areas impacted by a given natural hazard event. In this case, the process involved manually reviewing the location descriptions to determine the first-order administrative unit(s).

Coding Adaptation Actions by Hazard Type

To link the EM-DAT and CDP datasets, we coded each unique adaptation action in the CDP dataset according to the nine EM-DAT hazard types: drought, earthquake, extreme temperature, flood, landslide, mass movement, storm, volcanic activity, and wildfire. In this step, we only considered primary first-order effects. For example, while wildfires often coincide with droughts and heatwaves, we only consider wildfire-related adaptation actions to be those directly related to wildfire events. However, it is possible for one action to be directly related to multiple hazard types, or even to all-hazard types. Codes are binary; either a specific adaptation action is interpreted as being directly related to any given hazard event type (coded as '1') or it is coded as being unrelated to that specific event type ('0'). We include the full table of coded CDP adaptation actions below (Supplementary Table B1). We also include the results from an internal inter-coder reliability test to verify the coding scheme (Supplementary Figure B1). Lastly, in instances where actions were described in languages other than English, we used Google Translate to convert the texts into English prior to coding (action descriptions in Supplementary Table B1 are presented in the original language, as they appear in the CDP dataset).

[1] We drew on two publicly available sources of data for matching cities and first-order administrative units:
1) https://www.naturalearthdata.com/downloads/10m-cultural-vectors/10m-admin-1-states-provinces/
2) https://www.arcgis.com/home/item.html?id=9bc93fabc8d94747a22e811642ca426d

Supplementary Table 2: Coding individual adaptation actions descriptions reported to the CDP according to the type(s) of natural hazard each addresses.

Action No.	Adaptation Action Description	Drought	Earth-quake	Extreme Temp.	Flood	Land-slide	Mass Mov't	Storm	Volcanic Activity	Wild-fire	Generic	Other
1	Additional reservoirs and wells for water storage	1	0	0	1	0	0	1	0	0	0	0
2	Air quality initiatives	1	0	0	0	0	0	0	1	1	0	0
3	Awareness campaign/education to reduce water use	1	0	0	0	0	0	0	0	0	0	0
4	Biodiversity monitoring	0	0	0	0	0	0	0	0	0	0	1
5	Community engagement/education	0	0	0	0	0	0	0	0	0	0	1
6	Community engagement/education: Sea Level Rise Adaptation Plan	0	0	0	1	0	0	1	0	0	0	0
7	Cool pavement	0	0	1	0	0	0	0	0	0	0	0
8	Cooling centers, pools, water parks/plazas	0	0	1	0	0	0	0	0	0	0	0
9	Cooling systems for critical infrastructure	0	0	1	0	0	0	0	0	0	0	0
10	Crisis management including warning and evacuation systems	0	0	0	0	0	0	0	0	0	1	0
11	Crisis management including warning and evacuation systems: Heat Distribution Mapping	0	0	1	0	0	0	0	0	0	0	0
12	Disease prevention measures	0	0	0	0	0	0	0	0	0	0	1
13	Diversification of water supply	1	0	0	1	0	0	0	0	0	0	0
14	Diversifying power/energy supply	0	0	0	0	0	0	0	0	0	1	0
15	Economic diversification measures	0	0	0	0	0	0	0	0	0	0	1
16	Flood defences â€“ development and operation & storage	0	0	0	1	0	0	0	0	0	0	0
17	Flood mapping	0	0	0	1	0	0	0	0	0	0	0
18	Green roofs/walls	0	0	1	0	0	0	1	0	0	0	0
19	Hazard resistant infrastructure design and construction	0	1	1	1	0	0	1	0	0	0	0
20	Heat mapping and thermal imaging	0	0	1	0	0	0	0	0	0	0	0
21	Improve water supply distribution method	0	0	0	0	0	0	0	0	0	1	0
22	Incorporating climate change into long-term planning documents	1	0	1	1	0	0	1	0	1	0	0
23	Landslide risk mapping	0	0	0	0	1	0	0	0	0	0	0
24	Maintenance/repair â€“ leaking infrastructure	1	0	1	1	0	0	1	0	0	0	0
25	Nature based solutions for water	0	0	0	0	0	0	0	0	0	0	1
26	Other	0	0	0	0	0	0	0	0	0	0	1
27	Other: Communication campaign on climatic hazards	1	0	1	1	0	0	1	0	1	0	0

28	Other: environmental campaign	0	0	0	0	0	0	0	0	0	0	1
29	Other: establishment of the concept of water-friendly, situ-based cities, Situ Front City	0	0	0	0	0	0	0	0	0	0	1
30	Other: Treating wastewater for irrigation of green areas	0	0	0	0	0	0	0	0	0	0	1
31	Other: aÃ§Ã£o emergencial de contenÃ§Ã£o da linha da costa com enrocamento e paliÃ§adas.	0	0	0	1	0	0	1	0	0	0	0
32	Other: Acciones preventivas de incendios	0	0	0	0	0	0	0	0	1	0	0
33	Other: AdequaÃ§Ã£o de medidas	0	0	0	0	0	0	0	0	0	0	1
34	Other: Air Resistance	0	0	0	0	0	0	0	0	0	0	1
35	Other: Alien invasive species management	0	0	0	0	0	0	0	0	0	0	1
36	Other: AmpliaÃ§Ã£o e melhoria da rede de drenagem, limpeza de valas e rios.	0	0	0	1	0	0	1	0	0	0	0
37	Other: AprovaÃ§Ã£o de Leis especÃficas	0	0	0	0	0	0	0	0	0	0	1
38	Other: Automatic gates at flood prone roadways	0	0	0	1	0	0	0	0	0	0	0
39	Other: Back up power for traffic lights	0	0	0	0	0	0	0	0	0	1	0
40	Other: Basement Flood Protection	0	0	0	1	0	0	0	0	0	0	0
41	Other: Basic sanitation	0	0	0	0	0	0	0	0	0	0	1
42	Other: BMP Research and Implementation	0	0	0	0	0	0	0	0	0	0	1
43	Other: Broaden the slate of summer recreational	0	0	0	0	0	0	0	0	0	0	1
44	Other: CampaÃ±a de prevenciÃ³n y concientizaciÃ³n para tomar medidas de prevenciÃ³n contra la proliferaciÃ³n de insectos propagadores de enfermedades, ademÃ¡s de nebulizaciones en contra de los mosquitos, principalmente en colonias cercanas a los rÃos.	0	0	0	0	0	0	0	0	0	0	1
45	Other: Catchment flood risk management	0	0	0	1	0	0	0	0	0	0	0
46	Other: City Operations	0	0	0	0	0	0	0	0	0	0	1
47	Other: Climate Action Plan Implementation	1	0	1	1	0	0	1	0	1	0	0
48	Other: Climate Plan describes diferent accions	1	0	1	1	0	0	1	0	1	0	0
49	Other: Climate Plan describes some actions	1	0	1	1	0	0	1	0	1	0	0
50	Other: Climate Plan discribes diferent accions	1	0	1	1	0	0	1	0	1	0	0
51	Other: Coastal change adaptation	0	0	0	1	0	0	1	0	0	0	0
52	Other: Community Awareness/city health dept.	0	0	0	0	0	0	0	0	0	0	1
53	Other: ConservaciÃ³n de Ã¡reas naturales	0	0	0	0	0	0	0	0	0	0	1

54	Other: ConservaciÃ³n de suelos y prevenciÃ³n de inundaciones	0	0	0	1	1	0	0	0	0	0	0
55	Other: Control de plagas	0	0	0	0	0	0	0	0	0	0	1
56	Other: Controlled burnoffs and firebreaks	0	0	0	0	0	0	0	0	1	0	0
57	Other: CriaÃ§Ã£o da Defesa Civil e Corpo de Bombeiro	0	0	0	0	0	0	0	0	1	0	0
58	Other: Culvert Monitoring and Maintenance	0	0	0	1	0	0	1	0	0	0	0
59	Other: Declogging of water ways	0	0	0	1	0	0	1	0	0	0	0
60	Other: Decrease incidence of fire through hazard mitigation strategies	0	0	0	0	0	0	0	0	1	0	0
61	Other: Defensible Space Improvements	0	0	0	0	0	0	0	0	0	0	1
62	Other: DefiniciÃ³n de protocolos y responsabilidades para los entes de respuesta	0	0	0	0	0	0	0	0	0	1	0
63	Other: Desarrollo e implementaciÃ³n del Programa Regional de Manejo de Fuego para los municipios que integran Aipromades Lago de Chapala	0	0	0	0	0	0	0	0	1	0	0
64	Other: Desiliting of drains, expansion of drainages and canals	0	0	0	1	0	0	1	0	0	0	0
65	Other: DevelopÂ aÂ ClimateÂ SensitiveÂ &Â EnergyÂ EfficientÂ InfrastructureÂ usingÂ GreenÂ EngineeringÂ andÂ IncreaseÂ OpenÂ SpacesÂ andÂ Pedestrianization.Â	0	0	1	0	0	0	0	0	0	0	0
66	Other: Development of a Hazard Mitigation Plan	0	0	0	0	0	0	0	0	0	1	0
67	Other: Development Permit Areas	0	0	0	0	0	0	0	0	0	0	1
68	Other: Downscaling/Modeling of precipitation data	1	0	0	1	0	0	1	0	0	0	0
69	Other: Drainage and Stormwater Master Plan	0	0	0	1	0	0	1	0	0	0	0
70	Other: ECAP implementation	0	0	0	0	0	0	0	0	0	0	1
71	Other: Emergency Operations	0	0	0	0	0	0	0	0	0	1	0
72	Other: empleo de Bombas Antigranizo	0	0	0	0	0	0	1	0	0	0	0
73	Other: En caso de los riesgos relacionados con la adpatacion del cambio climatico se planea apoyar a las familias de escasos recursos por perdidas agricolas o de infraestructura	1	0	1	1	0	0	1	0	1	0	0
74	Other: Energy Efficiency and Passive Design	0	0	1	0	0	0	0	0	0	0	0
75	Other: Energy Resiliency	0	0	0	0	0	0	0	0	0	1	0
76	Other: Enhanced pest management	0	0	0	0	0	0	0	0	0	0	1
77	Other: Ensure quality/quantity drinking water	0	0	0	0	0	0	0	0	0	1	0

78	Other: Erosion Monitoring	0	0	0	0	1	0	0	0	0	0	0
79	Other: Examination of historical data	0	0	0	0	0	0	0	0	0	1	0
80	Other: Extreme Weather Operations Plan	0	0	1	1	0	0	1	0	0	0	0
81	Other: Facilitar acceso al agua	0	0	0	0	0	0	0	0	0	1	0
82	Other: Financial incentive, pervious surface reduction	0	0	0	1	0	0	0	0	0	0	0
83	Other: Fire Hazards have been mapped.	0	0	0	0	0	0	0	0	1	0	0
84	Other: Flood impact study	0	0	0	1	0	0	0	0	0	0	0
85	Other: Flood protection	0	0	0	1	0	0	0	0	0	0	0
86	Other: Food system resilience	0	0	0	0	0	0	0	0	0	0	1
87	Other: Forest Health and Management	0	0	0	0	0	0	0	0	0	0	1
88	Other: Forest management	0	0	0	0	0	0	0	0	0	0	1
89	Other: Forest management and restoration	0	0	0	0	0	0	0	0	0	0	1
90	Other: Fortalecimiento de brigadas forestales	0	0	0	0	0	0	0	0	0	0	1
91	Other: Funding Source for Clean Energy	0	0	0	0	0	0	0	0	0	0	1
92	Other: Governance reorganization	0	0	0	0	0	0	0	0	0	0	1
93	Other: Habitat projects that reduce flooding	0	0	0	1	0	0	0	0	0	0	0
94	Other: hail protection	0	0	0	0	0	0	1	0	0	0	0
95	Other: Harvest rainwater	1	0	0	0	0	0	0	0	0	0	0
96	Other: Hazard Disclosure Policy	0	0	0	0	0	0	0	0	0	1	0
97	Other: Hazardous fuel mitigation	0	0	0	0	0	0	0	0	1	0	0
98	Other: Heat Relief for Residents	0	0	1	0	0	0	0	0	0	0	0
99	Other: Heath action plan	0	0	0	0	0	0	0	0	0	0	1
100	Other: High efficiency street sweepers	0	0	0	0	0	0	0	0	0	0	1
101	Other: Home elevations above flood elevation	0	0	0	1	0	0	0	0	0	0	0
102	Other: IMPROVEMENT OF NATURAL DISASTER PREVENTION AND PROSPERITY	0	0	0	0	0	0	0	0	0	1	0
103	Other: Incorporating climate change into governance and administration policies	1	0	1	1	0	0	1	0	1	0	0
104	Other: Incorporating climate change into long-term planning documents	1	0	1	1	0	0	1	0	1	0	0
105	Other: Incorporating NFPA-1 regulations to historic burn areas	0	0	0	0	0	0	0	0	1	0	0
106	Other: Increase capacity (fire stations)	0	0	0	0	0	0	0	0	1	0	0
107	Other: Increase resilience of infrast. & build.	0	1	0	1	0	0	1	0	0	0	0
108	Other: Increase stability of river banks	0	0	0	1	0	0	0	0	0	0	0
109	Other: Increased Cross-Departmental Review	0	0	0	0	0	0	0	0	0	0	1
110	Other: Increased maintenance	0	0	0	0	0	0	0	0	0	0	1
111	Other: Increasing biodiversity in agriculture	0	0	0	0	0	0	0	0	0	0	1

112	Other: Increasing sustainable drainage and natural flood management measures across London	0	0	0	1	0	0	0	0	0	0	0
113	Other: IndicaÃ§Ã£o de espÃ©cies arbÃ³reas	0	0	0	0	0	0	0	0	0	0	1
114	Other: InformaciÃ³n desde el Ã¡rea de salud	0	0	0	0	0	0	0	0	0	0	1
115	Other: Install back-up generators and/or storage for critical facilities (and incorporate renewable energy where feasible)	0	0	0	0	0	0	0	0	0	1	0
116	Other: Installation of smart meters	0	0	0	0	0	0	0	0	0	0	1
117	Other: Integrated Stormwater Management Plan	0	0	0	1	0	0	1	0	0	0	0
118	Other: Joining Seawall Coalition	0	0	0	1	0	0	1	0	0	0	0
119	Other: land and subsidance mapping and research	0	0	0	0	1	1	0	0	0	0	0
120	Other: Les reboisements, la crÃ©ation des zones forestiÃ¨res tampons, les Haies vives autour des retenues d'eau	0	0	0	0	0	0	0	0	0	0	1
121	Other: Limpieza de canales, sumideros y quebradas	0	0	0	1	0	0	1	0	0	0	0
122	Other: Lucha antigranizo	0	0	0	0	0	0	1	0	0	0	0
123	Other: maintenance of public green	0	0	0	0	0	0	0	0	0	0	1
124	Other: managing alerts for citizens	0	0	0	0	0	0	0	0	0	1	0
125	Other: Managing flammable undergrowth in public and private at-risk areas	0	0	0	0	0	0	0	0	1	0	0
126	Other: Mangrove Planting	0	0	0	1	0	0	1	0	0	0	0
127	Other: Mantenimiento de Ã• reas Naturales Protegidas	0	0	0	0	0	0	0	0	0	0	1
128	Other: Mapa de sequÃas.	1	0	0	0	0	0	0	0	0	0	0
129	Other: mapping SuDS opportunities	0	0	0	1	0	0	1	0	0	0	0
130	Other: Mecanismo de compensaciÃ³n por pÃ©rdidas econÃ³micas - Seguros	0	0	0	0	0	0	0	0	0	0	1
131	Other: Mejora Infraestructura de Desague	0	0	0	1	0	0	1	0	0	0	0
132	Other: Mejora infraestructura de desagues	0	0	0	1	0	0	1	0	0	0	0
133	Other: Mejoramiento de desagÃ¼es pluviales	0	0	0	1	0	0	1	0	0	0	0
134	Other: Mesures de gestion de l'eau de pluie	0	0	0	1	0	0	1	0	0	0	0
135	Other: Meteorological warning systems	0	0	0	0	0	0	1	0	0	0	0
136	Other: Mitigate heat islands	0	0	1	0	0	0	0	0	0	0	0
137	Other: Mobile barriers - MOSE	0	0	0	1	0	0	0	0	0	0	0
138	Other: Modeling, Planning, and Implementation Action Development	0	0	0	0	0	0	0	0	0	0	1
139	Other: Monitoreo y Seguimiento	0	0	0	0	0	0	0	0	0	0	1
140	Other: Monitoring and Land Management	0	0	0	0	0	0	0	0	0	0	1

141	Other: Multiple Actions	0	0	0	0	0	0	0	0	0	0	1
142	Other: National Water Act & Coastal Plans	1	0	0	1	0	0	1	0	0	0	0
143	Other: New lock in Project Slussen (see above)	0	0	0	1	0	0	0	0	0	0	0
144	Other: Office of Emergency Management has set up a network of cooling stations throughout the city.	0	0	1	0	0	0	0	0	0	0	0
145	Other: Overheating risk assessments	0	0	1	0	0	0	0	0	0	0	0
146	Other: Pesquisa/estudo sobre erosÃ£o costeira	0	0	0	1	0	0	1	0	0	0	0
147	Other: Pest management and resilient trees	0	0	0	0	0	0	0	0	0	0	1
148	Other: Plan de Emergencia Comunal	0	0	0	0	0	0	0	0	0	1	0
149	Other: Planes de manejo ambiental	0	0	0	0	0	0	0	0	0	0	1
150	Other: PlanificaciÃ³n territorial para zonas que pueden ser impactadas por aumento del nivel del mar	0	0	0	1	0	0	0	0	0	0	0
151	Other: Planning	0	0	0	0	0	0	0	0	0	0	1
152	Other: Planning documents	0	0	0	0	0	0	0	0	0	0	1
153	Other: Plano de ContingÃªncia	0	0	0	0	0	0	0	0	0	1	0
154	Other: Plano Municipal de AcÃ§Ã£o	0	0	0	0	0	0	0	0	0	0	1
155	Other: Policy and Regulations	0	0	0	0	0	0	0	0	0	0	1
156	Other: Post-disaster energy security	0	0	0	0	0	0	0	0	0	1	0
157	Other: PrevenciÃ³n de deslizamientos	0	0	0	0	1	0	0	0	0	0	0
158	Other: Prevent saline intrusion	0	0	0	1	0	0	1	0	0	0	0
159	Other: Preventive inspection and monitoring, environmental education	0	0	0	0	0	0	0	0	0	1	0
160	Other: Proactive tree management to prevent impacts to overhead utilities during ice storm events.	0	0	0	0	0	0	1	0	0	0	0
161	Other: Programa de manejo del fuego	0	0	0	0	0	0	0	0	1	0	0
162	Other: Programa Regional de Manejo del Fuego	0	0	0	0	0	0	0	0	1	0	0
163	Other: Promoting thecniques and interventions aimed at making agriculture more resilient	0	0	0	0	0	0	0	0	0	0	1
164	Other: Promover a infiltraÃ§Ã£o da Ã¡gua	0	0	0	1	0	0	1	0	0	0	0
165	Other: ProtecciÃ³n comunidades vulnerables	0	0	0	0	0	0	0	0	0	0	1
166	Other: Protezione civile (gestione allerte)	0	0	0	0	0	0	0	0	0	1	0
167	Other: Protezione civile intercomunale, presso l'Unione Comuni montana Colline Metallifere. Il Centro affianca i singoli Comuni nella gestione delle emergenze,	0	0	0	0	0	0	0	0	0	1	0

	organizza il servizio di reperibilitÃ unificato e cura la formazione e lâ€™esercitazione del personale, garantisce infine la gestione unitaria della cosiddetta post emergenza. La ComunitÃ Montana garantisce la pronta reperibilitÃ di tecnici, operai e mezzi in caso di emergenze integrandosi con le strutture comunali. Le principali emergenze individuate riguardano gli eventi meteorologici, i dissesti idrogeologici, gli incendi e i terremoti.											
168	Other: Proyecto: control de inundaciones que contempla todas las subcuencas del distrito	0	0	0	1	0	0	0	0	0	0	0
169	Other: Public transport services	0	0	0	0	0	0	0	0	0	0	1
170	Other: radar intalled on waterbusses	0	0	0	0	0	0	1	0	0	0	0
171	Other: Raising shorelines citywide	0	0	0	1	0	0	0	0	0	0	0
172	Other: ReducciÃ³n de la Vulnerabilidad de la poblaciÃ³n	0	0	0	0	0	0	0	0	0	0	1
173	Other: Reduce Farm Runoff	0	0	0	0	0	0	0	0	0	0	1
174	Other: Reduce risk and impacts from sea level rise	0	0	0	1	0	0	1	0	0	0	0
175	Other: Reduce risks of damage from extreme events	0	0	0	0	0	0	0	0	0	1	0
176	Other: ReforestaciÃ³n en lugares con erosiÃ³n.	0	0	0	0	1	0	0	0	0	0	0
177	Other: Regional collaboration, levee management, and flood risk monitoring	0	0	0	1	0	0	0	0	0	0	0
178	Other: RenaturalizaÃ§Ã£o do Rio JacarÃ©	0	0	0	1	0	0	0	0	0	0	0
179	Other: ReorganizaÃ§Ã£o do Sistema de Macrodrenagem municipal	0	0	0	1	0	0	1	0	0	0	0
180	Other: Resilient species	0	0	0	0	0	0	0	0	0	0	1
181	Other: RevisiÃ³n y mejoramiento cableado publico	0	0	0	0	0	0	0	0	0	1	0
182	Other: Rice Seed Banking for Local Farmers	0	0	0	0	0	0	0	0	0	0	1
183	Other: Set-Back Lines, Coastal Management lines	0	0	0	1	0	0	1	0	0	0	0
184	Other: Several actions accross the Climate Plan	1	0	1	1	0	0	1	0	1	0	0
185	Other: Several actions across the Climate Plan	1	0	1	1	0	0	1	0	1	0	0
186	Other: Several actions against heat waves across the Climate Plan	0	0	1	0	0	0	0	0	0	0	0

187	Other: Several of above	0	0	0	0	0	0	0	0	0	0	1
188	Other: shelters	0	1	0	1	0	0	1	1	1	0	0
189	Other: Sistema de Infraestrutura-verde	0	0	0	0	0	0	0	0	0	0	1
190	Other: Sistemas de infraestutura-verde	0	0	0	0	0	0	0	0	0	0	1
191	Other: Snow clearing policy	0	0	0	0	0	1	1	0	0	0	0
192	Other: Step up comprehensive pest control	0	0	0	0	0	0	0	0	0	0	1
193	Other: Storm Water & Sanitary Master Plan	0	0	0	1	0	0	1	0	0	0	0
194	Other: Storm Water Management Planning, conservation promos and rebates for rain barrels/low-flow fixtures, drought-tolerant and naturalized park space conversions, non-potable irrigation, and capacity expansion as part of long term infrastructure planning	1	0	0	1	0	0	1	0	0	0	0
195	Other: Storm Water Master Plan	0	0	0	1	0	0	1	0	0	0	0
196	Other: Stormwater Management Plan, permits	0	0	0	1	0	0	1	0	0	0	0
197	Other: Stream Restoration and Park Infrastructure Improvement	0	0	0	1	0	0	0	0	0	0	0
198	Other: Subsidy for basement flood reduction measures	0	0	0	1	0	0	0	0	0	0	0
199	Other: SuDS guidance for sectors	0	0	0	1	0	0	1	0	0	0	0
200	Other: sustainable regeneration plan in coastal environment	0	0	0	1	0	0	1	0	0	0	0
201	Other: TÃ©cnicas nuevas para la reducciÃ³n de riesgo de incendio	0	0	0	0	0	0	0	0	1	0	0
202	Other: Temperaturas elevadas / Ondas de calor FenÃ³menos extremos - ProteÃ§Ãµes contra inundaÃ§Ãµes, Plantio de Ã¡rvores e/ou criaÃ§Ã£o de Ã¡reas verdes, Telhados e paredes ec	0	0	1	0	0	0	0	0	0	0	0
203	Other: The city purchased 16 new snow removal vehicles and is now using magnesium chloride mixed with salt for pretreatment.	0	0	0	0	0	1	1	0	0	0	0
204	Other: Transportation	0	0	0	0	0	0	0	0	0	0	1
205	Other: Tratamiento de aguas residuales	0	0	0	0	0	0	0	0	0	0	1
206	Other: Tratamiento de Residuos PatolgÃ³gicos	0	0	0	0	0	0	0	0	0	0	1
207	Other: tree height regulation in the green lane and replacement of brittle trees (easily fallen)	1	0	0	0	0	0	1	0	0	0	0
208	Other: Tree maintnenace/replacement program	1	0	0	0	0	0	1	0	0	0	0
209	Other: Tree monitoring and maintenance	1	0	0	0	0	0	1	0	0	0	0

210	Other: Tree planting and/or creation of green space, Technical inspection of cars, renewal of Municipal transport, Adopting the municipal Waste Management action Plan and Strategy.	0	0	1	0	1	0	1	0	0	0	0
211	Other: Update Standards and Regulations	0	0	0	0	0	0	0	0	0	0	1
212	Other: Upgrade stormwater infrastructure, Create a comprehensive municipal emergency plan	0	0	0	1	0	0	1	0	0	0	0
213	Other: Up-to-date code suites	0	0	0	0	0	0	0	0	0	0	1
214	Other: Urban Forestry to increase extent and health and diversity of the tree canopy	0	0	0	0	0	0	1	0	0	0	0
215	Other: Urban water spaces	1	0	1	0	0	0	0	0	0	0	0
216	Other: Use of Sustainable drainage systems	0	0	0	1	0	0	1	0	0	0	0
217	Other: Water conservation programs are in place. The City also has long range plans to improve and diversify the	1	0	0	0	0	0	0	0	0	0	0
218	Other: water coservation planning	1	0	0	0	0	0	0	0	0	0	0
219	Other: Water supply, agriculture, and safety	0	0	0	0	0	0	0	0	0	0	1
220	Other: Wet weather runoff management	0	0	0	1	0	0	1	0	0	0	0
221	Projects and policies targeted at those most vulnerable	0	0	0	0	0	0	0	0	0	0	1
222	Promoting and incentivizing water efficiency	1	0	0	0	0	0	0	0	0	0	0
223	Promoting low flow technologies	1	0	0	0	0	0	0	0	0	0	0
224	Public preparedness (including practice exercises/drills)	0	0	0	0	0	0	0	0	0	1	0
225	Public preparedness (including practice exercises/drills): Mangrove Planting	0	0	0	1	0	0	1	0	0	0	0
226	Real time risk monitoring	0	0	0	0	0	0	0	0	0	1	0
227	Resilience and resistance measures for buildings	0	1	1	1	0	0	1	0	0	0	0
228	Restrict development in at risk areas	0	0	0	1	1	1	0	1	1	0	0
229	Retrofit of existing buildings	1	0	1	1	0	0	1	0	0	0	0
230	Sea level rise modelling	0	0	0	1	0	0	1	0	0	0	0
231	Shading in public spaces, markets	0	0	1	0	0	0	0	0	0	0	0
232	Soil retention strategies	1	0	0	1	0	0	1	0	0	0	0
233	Storm water capture systems	0	0	0	1	0	0	1	0	0	0	0
234	Testing/vaccination programmes for vector-borne disease	0	0	0	0	0	0	0	0	0	0	1
235	Tree planting and/or creation of green space	0	0	1	0	1	0	1	0	0	0	0
236	Water butts/rainwater capture	1	0	0	0	0	0	0	0	0	0	0
237	Water efficient equipment and appliances	1	0	0	0	0	0	0	0	0	0	0

238	Water extraction protection	1	0	0	0	0	0	0	0	0	0	0
239	Water smart metering	1	0	0	0	0	0	0	0	0	0	0
240	Water use audits	1	0	0	0	0	0	0	0	0	0	0
241	Water use restrictions and standards	1	0	0	0	0	0	0	0	0	0	0
242	White roofs	0	0	1	0	0	0	0	0	0	0	0
243	Xeriscapes â€“ low water landscaping design	1	0	0	0	0	0	0	0	0	0	0

Inter-coder reliability test of adaptation action type categorization

In order to verify the results of the coding scheme in Supplementary Table A1, we conducted an internal inter-coder reliability test. In the first step, JH coded all adaptation actions according to the four types. Next, JH selected 20 adaptation actions from the CDP dataset (see the bold-faced adaptation actions in Table A1 above). While all individual actions were identified at random using a random number generator in the R software environment, the process was “semi-random” to ensure a representative sample of the different types of adaptation actions. In the second step, JH provided the adaptation action descriptions and detailed coding instructions, and DN, MM, and CP coded the 20 actions according to the types of hazard events each action directly addresses. In the last step, we calculated the level of agreement among the three coders. There was, on average, over 70% agreement between all four coders (Figure A1) concerning the categorization of adaptation actions. We then discussed all issues of non-agreement, and the final coding for each action of non-agreement was determined by applying codes agreed by two of the three coders).

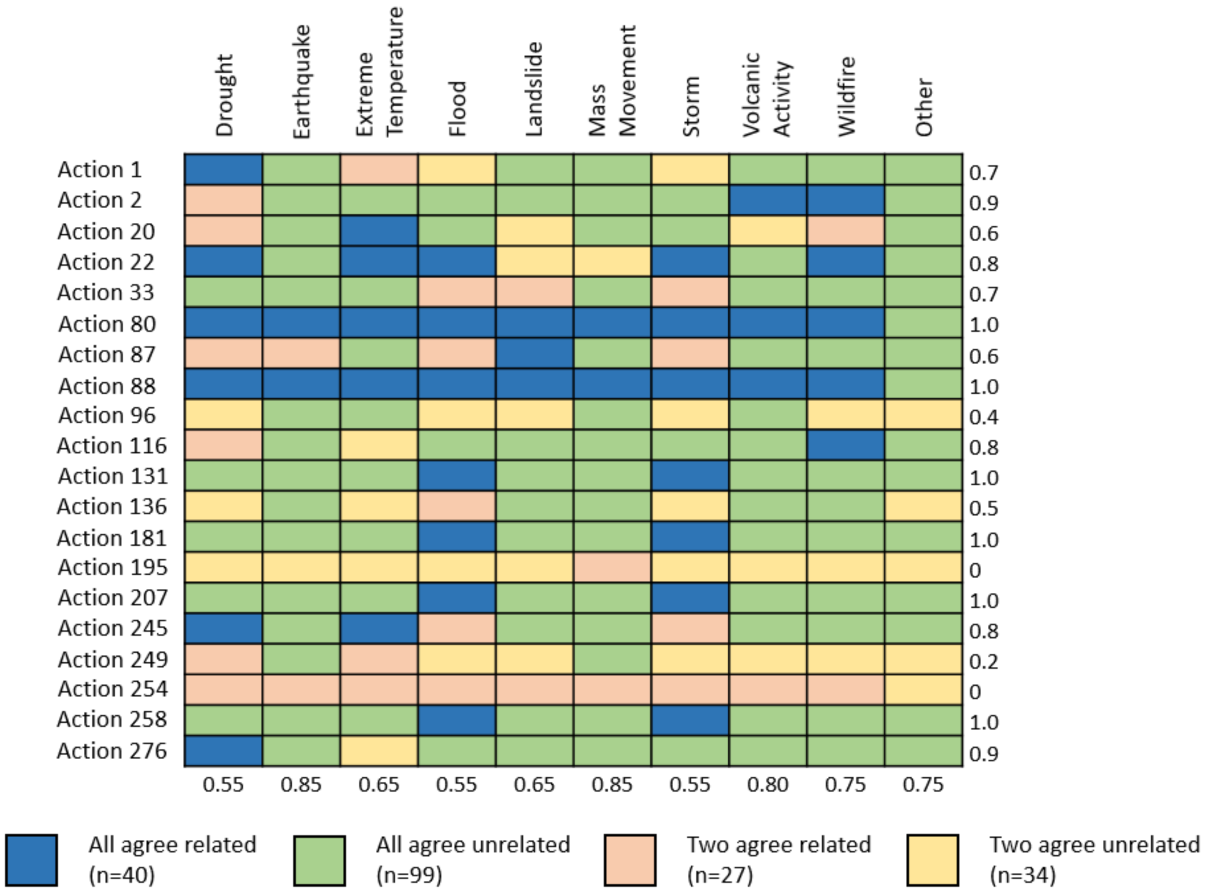

Supplementary Figure 2: Results from internal inter-coder reliability test for twenty semi-randomly selected adaptation actions.

Section 3: Dependent, Independent, and Control Variables

In this section, we provide the full descriptions of all variables investigated in the multiple regression analyses, including the data sources for each variable (Table B1). We provide additional discussion of the adaptive capacity variables, including the relevant theoretical foundations and justification for selecting these specific variables for inclusion in the analysis.

Variable descriptions

Supplementary Table 3: Variables included in multiple regression analyses.

VARIABLE	VARIABLE DESCRIPTION	SOURCE
Adaptation Action Type		
Specific	The number of adaptation actions reported by a city during 2018-2019 that were directly related to the same type of disaster events that occurred during 2013-2018.	CDP/EM-DAT
Expansive	The number of adaptation actions reported by a city during 2018-2019 that were not directly related to the type of disaster events (excluding "generic preparedness" and "other" actions, below) that occurred during 2013-2018.	CDP/EM-DAT
Generic preparedness	The number of adaptation actions reported by a city during 2018-2019 that consisted of measures to improve generic capacities for disaster response and management regardless of disaster event type.	CDP/EM-DAT
Other	The number of adaptation actions reported by a city during 2018-2019 that could not be classified as specific, expansive, or generic preparedness.	CDP/EM-DAT
Disaster Event Attributes (Raw Values)		
Event frequency*	The number of disaster events that occurred in a particular region (first-order administrative unit) during 2013-2018.	EM-DAT
Mean economic damages*	The average damages (in USD) to property, crops, and livestock incurred by all natural hazard events in a country during 2013-2018. The data for each year was adjusted in order to account for inflation using the Consumer Price Index (CPI), and standardized relative to the value of the U.S. dollar in 2021.	EM-DAT/CPI
Mean affected population*	The average number of individuals injured, or otherwise in need of assistance, as a result of all natural hazard events in a country during 2013-2018.	EM-DAT
Mean fatalities*	The average number of lives lost in all natural hazard events in a country during 2013-2018.	EM-DAT
Disaster Event Attributes (Baseline Values)		
Event frequency [baseline]	The three-year running average of the number of disaster events that occurred in a particular region (first-order administrative unit) during	EM-DAT
Mean economic damages [baseline]	The three-year running average of economic damages from all natural hazards in a given country in the EM-DAT dataset from 1998 to 2012. The data for each year was adjusted in order to account for inflation using the Consumer Price Index (CPI), and standardized relative to the value of the U.S. dollar in 2021.	EM-DAT/CPI
Mean affected population [baseline]	The three-year running average of individuals affected by all natural hazards in a given country in the EM-DAT dataset from 1998 to 2012.	EM-DAT
Mean fatalities [baseline]	The three-year running average of fatalities associated with all natural hazards in a given country in the EM-DAT dataset from 1998 to 2012.	EM-DAT
Adaptive Capacity		

Political stability	Variable "ffp_sl" from the Quality of Government (QoG) dataset, combining state performance in five areas: public confidence in the political process, political opposition (peaceful demonstrations and riots), transparency (evidence and considerations of corruption), openness and fairness of the political process (political rights, representative government, leadership transition, free elections), and political violence (political assassinations, armed insurgents, and terrorism). Note: we reverse the scale of this variable for ease of interpretation in the regression models, whereby high scores indicate greater stability.	QoG
Meritocracy	Variable "v2stcritrecadm" from the Varieties of Democracy (V-DEM) dataset indicating the extent to which appointment decisions within the state administration are based on skill and merit. High scores indicate greater reliance on skill and merit.	V-DEM
Stakeholder diversity	Variable "v2dlconslt" from the Varieties of Democracy (V-DEM) dataset indicating extent to which decisions are based on consultations involving actors from across the political spectrum and sectors of society. Higher values indicate greater diversity.	V-DEM
Local government power	Variable "v2xel_locelec" from the Varieties of Democracy (V-DEM) dataset indicating whether countries have elected local governments that are able to operate independently from unelected local actors. Higher values indicate greater independence.	V-DEM
Control Variables		
GDP per capita	Gross domestic product per capita from 2017.	World Bank
City population	The total urban population of a given city.	CDP
Time lag	The average number of years between natural hazard events and adaptation actions taken by a given city.	CDP/EM-DAT

*For some models (Supplementary Tables 1b-5b, 11b, 14b, and D16e-D16h), economic damages were normalized using Gross Domestic Product (GDP) per capita. Disaster frequency was normalized by the total number of disasters that occurred within the country over the time period of the study, while affected population and fatalities were normalized by city population.

Adaptive Capacity Control Variables

We included several relevant measures as controls for determinants of adaptive capacity, which is an aggregation of properties shaping the propensity or ability of any governing system to undertake adaptation actions. Due to the lack of comparable global data to determine the adaptive capacity of cities and local governments, we rely on national-level indicators of the preconditions for adaptation actions. Hence, the caveat is that these indicators may overlook information concerning local variation. The selection of measures is based on three premises.

First is the analytical separation of adaptation actions from the adaptive capacity of a system. In this study, adaptation actions refer to measures undertaken by a city to alleviate the adverse impacts of climate change and/or enhance resilience to natural hazard events. A number of system attributes are important preconditions that shape the ability of cities to formulate, initiate, and implement these adaptation actions.

Second, adaptation actions are defined here relatively narrowly as measures resulting from deliberate decisions by policy actors, including politicians, bureaucrats, and other stakeholders, to change the existing order. Therefore, for this study, we assume that only those system attributes that directly affect decisions concerning adaptation actions are relevant to consider in the models. These measures include different system attributes that support the collective ability of policy actors to translate the

experience of hazard events into adaptation actions. Other community features commonly associated with adaptive capacity, for instance, various social relationships and the ability of individuals to self-organize and innovate, are crucial elements of adaptive capacity, more generally but not relevant factors conditioning policy-making associated with adaptation actions.

Third, the literature identifies several important determinants of adaptive capacity related to attributes of individuals and the internal dynamics of public organizations, which in turn may enable or constrain policy-making concerning adaptation actions. Examples include institutional memory, the ability of policy actors to learn and change assumptions, and the vision and entrepreneurial skills of leaders. These attributes, however, are elusive, and to our knowledge, currently, no data exist to measure these across a wider set of cases. Hence there is a lack of data to enable comparisons of these attributes in cities around the world.

Following these premises, we set out from core dimensions identified by established frameworks of adaptive capacity and searched available datasets with global reach for the best possible proxy measures for each theme, respectively. To identify relevant data, we consulted two datasets: *Varieties of Democracy* (V-Dem)[1] and the *Quality of Government* (QoG)[2]. The selection of indicators has also been guided by the scope of each dataset, respectively, where some indicators were deemed relevant but excluded because data were only available for a limited number of the 58 countries included in the analysis. For example, the QoG dataset (p. 565) entails indicators of the quality of democracy, which recognize the essential role of democratic participation and oversight to learning and adaptation. Although these indicators would be relevant for the purpose of this study, data for these indicators were only available for 41 countries.

Political stability, including, e.g., the absence of civil conflict and the functioning of democracy[3,4], is depicted as a prerequisite for adaptation. Some studies[5] define political stability as a proxy measure indicating a willingness to invest in adaptation. Other work[6] emphasizes the importance of stable political institutions that provide predictability in support of collective action. Political stability may also interact with other factors associated with adaptive capacity. For example, a decrease in political stability may trigger consequent problems related to economic conditions, which, in turn, can constrain adaptation initiatives[7]. The current study measures political stability by an aggregated index of 'state legitimacy' derived from the Fragile States Index (https://fragilestatesindex.org/indicators/p1/) accessed via the Quality of Government dataset, combining state performance in five areas: public confidence in the political process, political opposition (peaceful demonstrations and riots), transparency (evidence and considerations of corruption), openness and fairness of the political process (political rights, representative government, leadership transition, free elections), and political violence (political assassinations, armed insurgents, and terrorism). For this study, we recoded the Fragile States Index indicator, which originally measures political stability on a scale 10-0 where higher scores indicate less stability, so that higher scores indicate greater stability.

Studies suggest that adaptive capacity is conditioned by *national development* since financial resources are needed to support the implementation of adaptation policy measures. In addition, various other resources (political, human, legal, and technological) condition institutional effectiveness, including the ability of institutions to change norms and rules[3,6,8–10]. Given that adaptive capacity is related to economic well-being, assessments of adaptive capacity use Gross National Income (GNI) as one indicator to demonstrate wealth[11]. On this basis, the current study includes GNI (data derived from the World Bank) as one proxy measure of adaptive capacity. It should be noted that GNI is indirectly included in one of the regression models (Supplementary Table 21b), where we used Gross Domestic

Product (GDP) per capita to normalize economic losses of disasters. Therefore, GNI was removed as a control from this model.

Another dimension of adaptive capacity considered in this work concerns *meritocracy*, specifically knowledge of individuals representing state bureaucracy. Knowledge is generally defined both as a determinant and indicator of the adaptive capacity of individuals, communities, organizations, and countries[4,12]. Given our interest in adaptation actions undertaken by cities as government entities, the study focuses on the role of knowledge in supporting policy-making. Specifically, we focus on the capacity of individuals to interpret information regarding current and future local environmental conditions as the basis for making policy decisions about adaptation actions[10]. To this end, we use a generic indicator of 'meritocracy' (derived from the V-Dem dataset), capturing whether appointment decisions within state administration are based on personal and political connections versus skill and merit. Thus, the assumption is that appointment decisions based on skill and merit help sustaining a cadre of bureaucrats and other policy professionals with the capacity to make informed decisions based on experience. The scale ranges 0-4, with higher values indicating greater reliance on skill and merit.

Stakeholder diversity, defined here as the participation and influence of multiple interests and actors in policy-making, is another determinant of adaptive capacity. Ensuring broad participation of actors representing organizations at different levels and sectors, including non-state actors, is a way to give room for multiple opinions, beliefs, and problem definitions and to widen the range of options in policy-making[6]. Such variety provides access to different types of knowledge[13] that reduce uncertainty and support learning and the development and implementation of adaptation measures[14]. To account for stakeholder diversity, we relied on information concerning the 'range of consultation' (derived from the V-Dem dataset), which measures the width of consultations at elite levels when important policy changes are considered. This scale ranges from 0 (indicating no consultation where a leader or small group make all decisions on their own) to 5 (decisions are based on consultations involving actors from across the political spectrum and sectors of society).

Finally, the study includes a measure of *local government power* as a means to control for whether adaptation actions are more frequent in cities located in countries with elected local governments that are able to operate independently from unelected local actors (with the exception of judicial bodies). The Intergovernmental Panel on Climate Change recognizes the importance of empowering local communities to take action for reducing vulnerability and strengthening resilience, including responsibility and decision-making to improve preparedness through post-disaster assessments[15]. While the literature[5] emphasizes the multifaceted nature of local-level adaptation (spanning broad sets of community characteristics and interactions), we focused exclusively on local government independence from the involvement of unelected local actors. Hence, the assumption is that greater independence facilitates adaptation actions, partially by avoiding or bypassing political conflict and power-struggles[16,17]. We include a measure of local government power (derived from the V-Dem dataset) ranging from 0 (countries have no elected local governments) to 1 (elected local governments operate without restrictions).

Section 4: Full Regression Results for All Types of Adaptation Actions

Tables 4-8: bivariate regression models of each type of adaptation action with respect to absolute hazard frequency and severity measures

Table 4: All actions with respect to hazard frequency and severity

	Dependent variable:			
	All actions			
	(1)	(2)	(3)	(4)
disasters	0.557**			
	(0.264)			
damages		0.588**		
		(0.239)		
affected			-0.569	
			(0.412)	
deaths				-0.164
				(0.313)
Constant	5.981***	5.992***	6.633***	6.696***
	(0.412)	(0.447)	(0.325)	(0.339)
Observations	549	470	507	510
R^2	0.008	0.013	0.004	0.001
Adjusted R^2	0.006	0.011	0.002	-0.001
Residual Std. Error	7.136 (df = 547)	7.257 (df = 468)	7.128 (df = 505)	7.152 (df = 508)
F Statistic	4.437** (df = 1; 547)	6.060** (df = 1; 468)	1.911 (df = 1; 505)	0.276 (df = 1; 508)
Note:				*p<0.1; **p<0.05; ***p<0.01

Table 5: Specific actions with respect to hazard frequency and severity

	Dependent variable:			
	Specific actions			
	(1)	(2)	(3)	(4)
disasters	0.615***			
	(0.143)			
damages		0.322**		
		(0.134)		
affected			-0.194	
			(0.219)	
deaths				-0.086
				(0.174)
Constant	2.436***	2.884***	3.118***	3.216***
	(0.223)	(0.250)	(0.173)	(0.189)
Observations	549	470	507	510
R^2	0.033	0.012	0.002	0.0005
Adjusted R^2	0.031	0.010	-0.0004	-0.001
Residual Std. Error	3.859 (df = 547)	4.065 (df = 468)	3.801 (df = 505)	3.980 (df = 508)
F Statistic	18.476*** (df = 1; 547)	5.782** (df = 1; 468)	0.782 (df = 1; 505)	0.244 (df = 1; 508)
Note:				*p<0.1; **p<0.05; ***p<0.01

Table 6: Expansive actions with respect to hazard frequency and severity

	Dependent variable:			
	Expansive actions			
	(1)	(2)	(3)	(4)
disasters	-0.231***			
	(0.069)			
damages		0.092		
		(0.059)		
affected			-0.070	
			(0.108)	
deaths				0.004
				(0.082)
Constant	1.379***	0.943***	1.109***	1.112***
	(0.108)	(0.110)	(0.086)	(0.089)
Observations	549	470	507	510
R^2	0.020	0.005	0.001	0.00000
Adjusted R^2	0.018	0.003	-0.001	-0.002
Residual Std. Error	1.864 (df = 547)	1.789 (df = 468)	1.878 (df = 505)	1.869 (df = 508)
F Statistic	11.187*** (df = 1; 547)	2.438 (df = 1; 468)	0.416 (df = 1; 505)	0.002 (df = 1; 508)
Note:				*p<0.1; **p<0.05; ***p<0.01

Table 7: Generic preparedness actions with respect to hazard frequency and severity

	Dependent variable:			
	General actions			
	(1)	(2)	(3)	(4)
disasters	0.067			
	(0.064)			
damages		0.103^{*}		
		(0.053)		
affected			-0.070	
			(0.097)	
deaths				-0.031
				(0.071)
Constant	0.977^{***}	0.909^{***}	1.066^{***}	1.020^{***}
	(0.100)	(0.100)	(0.076)	(0.077)
Observations	549	470	507	510
R^2	0.002	0.008	0.001	0.0004
Adjusted R^2	0.0002	0.006	-0.001	-0.002
Residual Std. Error	1.727 (df = 547)	1.622 (df = 468)	1.672 (df = 505)	1.613 (df = 508)
F Statistic	1.085 (df = 1; 547)	3.712^{*} (df = 1; 468)	0.530 (df = 1; 505)	0.194 (df = 1; 508)
Note:				*p<0.1; **p<0.05; ***p<0.01

Table 8: Other actions with respect to hazard frequency and severity

	Dependent variable:			
	Other actions			
	(1)	(2)	(3)	(4)
disasters	0.107			
	(0.077)			
damages		0.072		
		(0.072)		
affected			-0.235^{*}	
			(0.123)	
deaths				-0.051
				(0.093)
Constant	1.189^{***}	1.256^{***}	1.340^{***}	1.347^{***}
	(0.120)	(0.135)	(0.097)	(0.101)
Observations	549	470	507	510
R^2	0.003	0.002	0.007	0.001
Adjusted R^2	0.002	-0.00003	0.005	-0.001
Residual Std. Error	2.084 (df = 547)	2.187 (df = 468)	2.136 (df = 505)	2.125 (df = 508)
F Statistic	1.906 (df = 1; 547)	0.987 (df = 1; 468)	3.621^{*} (df = 1; 505)	0.297 (df = 1; 508)
Note:				*p<0.1; **p<0.05; ***p<0.01

Tables 9-13: bivariate regression models of each type of adaptation action with respect to normalized hazard frequency and severity measures

Table 9: All actions with respect to normalized hazard frequency and severity

	Dependent variable:			
	All actions			
	(1)	(2)	(3)	(4)
disasters	1.455			
	(1.422)			
damages		-0.526		
		(0.382)		
affected			-0.238^{*}	
			(0.139)	
deaths				-0.627^{**}
				(0.265)
Constant	6.159^{***}	6.964^{***}	6.640^{***}	6.887^{***}
	(0.506)	(0.380)	(0.321)	(0.330)
Observations	548	470	502	505
R^2	0.002	0.004	0.006	0.011
Adjusted R^2	0.0001	0.002	0.004	0.009
Residual Std. Error	7.163 (df = 546)	7.289 (df = 468)	7.142 (df = 500)	7.135 (df = 503)
F Statistic	1.047 (df = 1; 546)	1.893 (df = 1; 468)	2.948^{*} (df = 1; 500)	5.615^{**} (df = 1; 503)
Note:				*p<0.1; **p<0.05; ***p<0.01

Table 10: Specific actions with respect to normalized hazard frequency and severity

	Dependent variable:			
	Specific actions			
	(1)	(2)	(3)	(4)
disasters	1.102			
	(0.778)			
damages		-0.312		
		(0.214)		
affected			-0.119	
			(0.074)	
deaths				-0.248^{*}
				(0.148)
Constant	2.770^{***}	3.427^{***}	3.131^{***}	3.282^{***}
	(0.277)	(0.213)	(0.171)	(0.184)
Observations	548	470	502	505
R^2	0.004	0.005	0.005	0.006
Adjusted R^2	0.002	0.002	0.003	0.004
Residual Std. Error	3.920 (df = 546)	4.081 (df = 468)	3.809 (df = 500)	3.985 (df = 503)
F Statistic	2.003 (df = 1; 546)	2.126 (df = 1; 468)	2.614 (df = 1; 500)	2.808^{*} (df = 1; 503)
Note:				*p<0.1; **p<0.05; ***p<0.01

Table 11: Expansive actions with respect to normalized hazard frequency and severity

	Dependent variable:			
	Expansive actions			
	(1)	(2)	(3)	(4)
disasters	0.005			
	(0.374)			
damages		0.047		
		(0.094)		
affected			-0.051	
			(0.036)	
deaths				-0.165^{**}
				(0.069)
Constant	1.137^{***}	1.036^{***}	1.122^{***}	1.180^{***}
	(0.133)	(0.093)	(0.085)	(0.086)
Observations	548	470	502	505
R^2	0.00000	0.001	0.004	0.011
Adjusted R^2	-0.002	-0.002	0.002	0.009
Residual Std. Error	1.884 (df = 546)	1.793 (df = 468)	1.881 (df = 500)	1.865 (df = 503)
F Statistic	0.0002 (df = 1; 546)	0.252 (df = 1; 468)	1.983 (df = 1; 500)	5.655^{**} (df = 1; 503)
Note:				*p<0.1; **p<0.05; ***p<0.01

Table 12: Generic actions with respect to normalized hazard frequency and severity

	Dependent variable:			
	General actions			
	(1)	(2)	(3)	(4)
disasters	-0.037			
	(0.343)			
damages		-0.057		
		(0.085)		
affected			-0.018	
			(0.033)	
deaths				-0.068
				(0.060)
Constant	1.060***	1.063***	1.065***	1.037***
	(0.122)	(0.085)	(0.075)	(0.075)
Observations	548	470	502	505
R^2	0.00002	0.001	0.001	0.003
Adjusted R^2	-0.002	-0.001	-0.001	0.001
Residual Std. Error	1.730 (df = 546)	1.627 (df = 468)	1.678 (df = 500)	1.616 (df = 503)
F Statistic	0.012 (df = 1; 546)	0.451 (df = 1; 468)	0.317 (df = 1; 500)	1.277 (df = 1; 503)
Note:				*p<0.1; **p<0.05; ***p<0.01

Table 13: Other actions with respect to normalized hazard frequency and severity

	Dependent variable:			
	Other actions			
	(1)	(2)	(3)	(4)
disasters	0.385			
	(0.414)			
damages		-0.204*		
		(0.114)		
affected			-0.049	
			(0.042)	
deaths				-0.147*
				(0.079)
Constant	1.192***	1.439***	1.322***	1.388***
	(0.147)	(0.114)	(0.097)	(0.098)
Observations	548	470	502	505
R^2	0.002	0.007	0.003	0.007
Adjusted R^2	-0.0002	0.005	0.001	0.005
Residual Std. Error	2.088 (df = 546)	2.181 (df = 468)	2.148 (df = 500)	2.125 (df = 503)
F Statistic	0.864 (df = 1; 546)	3.170* (df = 1; 468)	1.367 (df = 1; 500)	3.486* (df = 1; 503)
Note:				*p<0.1; **p<0.05; ***p<0.01

Tables 14-18: Bivariate regression models of each type of adaptation action with respect to 15-year baseline hazard frequency and severity dummy variables

Table 14: All actions with respect to baseline frequency and severity

	Dependent variable:			
	All actions			
	(1)	(2)	(3)	(4)
disasters	1.055*			
	(0.612)			
damages		0.013		
		(0.700)		
affected			0.002	
			(0.666)	
deaths				-0.941
				(0.720)
Constant	5.992***	6.561***	6.564***	6.786***
	(0.451)	(0.355)	(0.366)	(0.349)
Observations	549	549	549	549
R^2	0.005	0.00000	0.00000	0.003
Adjusted R^2	0.004	-0.002	-0.002	0.001
Residual Std. Error (df = 547)	7.145	7.165	7.165	7.154
F Statistic (df = 1; 547)	2.970*	0.0004	0.00001	1.707
Note:	*p<0.1; **p<0.05; ***p<0.01			

Table 15: Specific actions with respect to baseline frequency and severity

	Dependent variable:			
	Specific actions			
	(1)	(2)	(3)	(4)
disasters	0.845**			
	(0.334)			
damages		-0.012		
		(0.383)		
affected			-0.236	
			(0.364)	
deaths				-0.378
				(0.395)
Constant	2.622***	3.083***	3.151***	3.169***
	(0.246)	(0.194)	(0.200)	(0.191)
Observations	549	549	549	549
R^2	0.012	0.00000	0.001	0.002
Adjusted R^2	0.010	-0.002	-0.001	-0.0001
Residual Std. Error (df = 547)	3.901	3.924	3.922	3.921
F Statistic (df = 1; 547)	6.391**	0.001	0.418	0.919
Note:	*p<0.1; **p<0.05; ***p<0.01			

Table 16: Expansive actions with respect to baseline frequency and severity

	Dependent variable:			
	Expansive actions			
	(1)	(2)	(3)	(4)
disasters	-0.174			
	(0.161)			
damages		-0.012		
		(0.184)		
affected			-0.023	
			(0.175)	
deaths				-0.087
				(0.189)
Constant	1.231***	1.140***	1.144***	1.157***
	(0.119)	(0.093)	(0.096)	(0.092)
Observations	549	549	549	549
R^2	0.002	0.00001	0.00003	0.0004
Adjusted R^2	0.0003	-0.002	-0.002	-0.001
Residual Std. Error (df = 547)	1.881	1.883	1.883	1.882
F Statistic (df = 1; 547)	1.167	0.004	0.017	0.213
Note:	*p<0.1; **p<0.05; ***p<0.01			

Table 17: Generic preparedness actions with respect to baseline frequency and severity

	Dependent variable:			
	General actions			
	(1)	(2)	(3)	(4)
disasters	0.102			
	(0.148)			
damages		-0.054		
		(0.169)		
affected			0.131	
			(0.161)	
deaths				-0.315*
				(0.173)
Constant	0.992***	1.061***	1.008***	1.121***
	(0.109)	(0.086)	(0.088)	(0.084)
Observations	549	549	549	549
R^2	0.001	0.0002	0.001	0.006
Adjusted R^2	-0.001	-0.002	-0.001	0.004
Residual Std. Error (df = 547)	1.728	1.729	1.728	1.724
F Statistic (df = 1; 547)	0.474	0.103	0.663	3.301*
Note:	*p<0.1; **p<0.05; ***p<0.01			

Table 18: Other actions with respect to baseline frequency and severity

	Dependent variable:			
	Other actions			
	(1)	(2)	(3)	(4)
disasters	0.282			
	(0.178)			
damages		0.092		
		(0.204)		
affected			0.130	
			(0.194)	
deaths				-0.160
				(0.210)
Constant	1.147***	1.277***	1.261***	1.338***
	(0.131)	(0.103)	(0.107)	(0.102)
Observations	549	549	549	549
R^2	0.005	0.0004	0.001	0.001
Adjusted R^2	0.003	-0.001	-0.001	-0.001
Residual Std. Error (df = 547)	2.083	2.087	2.087	2.086
F Statistic (df = 1; 547)	2.500	0.203	0.453	0.579
Note:	*p<0.1; **p<0.05; ***p<0.01			

Tables 19-23 contain bivariate regression models of each type of adaptation actions with respect to adaptive capacity control variables

Table 19: All actions with respect to adaptive capacity control variables

	Dependent variable:			
	All actions			
	(1)	(2)	(3)	(4)
political stability	0.688***			
	(0.127)			
meritocracy		1.599***		
		(0.346)		
stakeholder diversity			0.214	
			(0.351)	
local gov. power				0.369*
				(0.212)
Constant	2.571***	4.619***	6.322***	3.231*
	(0.797)	(0.522)	(0.533)	(1.960)
Observations	538	546	546	539
R^2	0.052	0.038	0.001	0.006
Adjusted R^2	0.050	0.036	-0.001	0.004
Residual Std. Error	7.017 (df = 536)	7.040 (df = 544)	7.174 (df = 544)	7.193 (df = 537)
F Statistic	29.472*** (df = 1; 536)	21.307*** (df = 1; 544)	0.372 (df = 1; 544)	3.046* (df = 1; 537)
Note:				*p<0.1; **p<0.05; ***p<0.01

Table 20: Specific actions with respect to adaptive capacity control variables

	Dependent variable:			
	Specific actions			
	(1)	(2)	(3)	(4)
political stability	0.428***			
	(0.069)			
meritocracy		1.090***		
		(0.188)		
stakeholder diversity			-0.070	
			(0.192)	
local gov. power				0.178
				(0.116)
Constant	0.618	1.755***	3.184***	1.484
	(0.433)	(0.283)	(0.292)	(1.074)
Observations	538	546	546	539
R^2	0.067	0.058	0.0002	0.004
Adjusted R^2	0.066	0.057	-0.002	0.003
Residual Std. Error	3.812 (df = 536)	3.811 (df = 544)	3.928 (df = 544)	3.941 (df = 537)
F Statistic	38.675*** (df = 1; 536)	33.800*** (df = 1; 544)	0.132 (df = 1; 544)	2.362 (df = 1; 537)
Note:				*p<0.1; **p<0.05; ***p<0.01

Table 21: Expansive actions with respect to adaptive capacity control variables

	Dependent variable:			
	Expansive actions			
	(1)	(2)	(3)	(4)
political stability	0.073**			
	(0.034)			
meritocracy		0.086		
		(0.093)		
stakeholder diversity			0.264***	
			(0.092)	
local gov. power				0.111**
				(0.056)
Constant	0.713***	1.033***	0.812***	0.121
	(0.214)	(0.140)	(0.139)	(0.515)
Observations	538	546	546	539
R^2	0.008	0.002	0.015	0.007
Adjusted R^2	0.007	-0.0003	0.013	0.006
Residual Std. Error	1.885 (df = 536)	1.886 (df = 544)	1.873 (df = 544)	1.889 (df = 537)
F Statistic	4.558** (df = 1; 536)	0.860 (df = 1; 544)	8.290*** (df = 1; 544)	4.003** (df = 1; 537)
Note:				*p<0.1; **p<0.05; ***p<0.01

Table 22: Generic preparedness actions with respect to adaptive capacity control variables

	Dependent variable:			
	General actions			
	(1)	(2)	(3)	(4)
political stability	0.087***			
	(0.031)			
meritocracy		0.167**		
		(0.085)		
stakeholder diversity			-0.019	
			(0.085)	
local gov. power				0.053
				(0.051)
Constant	0.517***	0.841***	1.069***	0.569
	(0.194)	(0.128)	(0.129)	(0.474)
Observations	538	546	546	539
R^2	0.014	0.007	0.0001	0.002
Adjusted R^2	0.013	0.005	-0.002	0.0001
Residual Std. Error	1.707 (df = 536)	1.725 (df = 544)	1.731 (df = 544)	1.738 (df = 537)
F Statistic	7.875*** (df = 1; 536)	3.859** (df = 1; 544)	0.049 (df = 1; 544)	1.076 (df = 1; 537)
Note:				*p<0.1; **p<0.05; ***p<0.01

Table 23: Other actions with respect to adaptive capacity control variables

	Dependent variable:			
	Other actions			
	(1)	(2)	(3)	(4)
political stability	0.101***			
	(0.038)			
meritocracy		0.256**		
		(0.102)		
stakeholder diversity			0.039	
			(0.102)	
local gov. power				0.027
				(0.062)
Constant	0.724***	0.991***	1.258***	1.058*
	(0.237)	(0.154)	(0.155)	(0.571)
Observations	538	546	546	539
R^2	0.013	0.011	0.0003	0.0004
Adjusted R^2	0.011	0.010	-0.002	-0.002
Residual Std. Error	2.091 (df = 536)	2.080 (df = 544)	2.091 (df = 544)	2.095 (df = 537)
F Statistic	7.093*** (df = 1; 536)	6.258** (df = 1; 544)	0.145 (df = 1; 544)	0.191 (df = 1; 537)
Note:				*p<0.1; **p<0.05; ***p<0.01

Tables 24-26 contain multiple regression models of each type of adaptation action with respect to absolute, normalized, and baseline hazard frequency and severity measures, respectively, and do not include any additional control variables.

Table 24: Adaptation action types with respect to hazard frequency and severity

	Dependent variable:				
	All	Specific	Expansive	General	Other
	(1)	(2)	(3)	(4)	(5)
disasters	0.081	0.373**	-0.307***	0.008	0.006
	(0.324)	(0.176)	(0.080)	(0.073)	(0.101)
damages	0.867***	0.342**	0.242***	0.132**	0.152*
	(0.295)	(0.160)	(0.073)	(0.066)	(0.092)
affected	-0.618	-0.182	-0.131	-0.063	-0.240*
	(0.455)	(0.247)	(0.112)	(0.102)	(0.142)
deaths	-0.236	-0.106	-0.042	-0.042	-0.046
	(0.342)	(0.185)	(0.084)	(0.077)	(0.106)
Constant	5.737***	2.452***	1.165***	0.899***	1.222***
	(0.539)	(0.292)	(0.132)	(0.121)	(0.168)
Observations	446	446	446	446	446
R^2	0.029	0.039	0.041	0.013	0.013
Adjusted R^2	0.020	0.030	0.032	0.004	0.004
Residual Std. Error (df = 441)	7.130	3.867	1.752	1.599	2.221
F Statistic (df = 4; 441)	3.289**	4.432***	4.659***	1.418	1.485
Note:			*p<0.1; **p<0.05; ***p<0.01		

Table 25: Adaptation action types with respect to normalized frequency and severity

	Dependent variable:				
	All	Specific	Expansive	General	Other
	(1)	(2)	(3)	(4)	(5)
disasters	1.584	0.977	-0.665	0.608	0.664
	(1.909)	(1.045)	(0.472)	(0.426)	(0.591)
damages	-0.134	-0.077	0.052	0.004	-0.114
	(0.444)	(0.243)	(0.110)	(0.099)	(0.138)
affected	-0.312	-0.125	-0.087	-0.038	-0.061
	(0.264)	(0.145)	(0.065)	(0.059)	(0.082)
deaths	-0.606**	-0.257*	-0.137**	-0.071	-0.141
	(0.282)	(0.154)	(0.070)	(0.063)	(0.087)
Constant	6.585***	3.133***	1.266***	0.903***	1.283***
	(0.689)	(0.377)	(0.170)	(0.154)	(0.213)
Observations	441	441	441	441	441
R^2	0.018	0.012	0.018	0.010	0.014
Adjusted R^2	0.009	0.003	0.009	0.001	0.005
Residual Std. Error (df = 436)	7.194	3.938	1.779	1.607	2.228
F Statistic (df = 4; 436)	1.988*	1.338	1.953	1.057	1.569
Note:			*p<0.1; **p<0.05; ***p<0.01		

Table 26: Adaptation action types with respect to baseline control variables

	Dependent variable:				
	All	Specific	Expansive	General	Other
	(1)	(2)	(3)	(4)	(5)
disasters	1.267**	0.980***	-0.167	0.140	0.314*
	(0.629)	(0.343)	(0.166)	(0.152)	(0.183)
damages	0.359	0.197	0.00003	0.014	0.148
	(0.734)	(0.401)	(0.194)	(0.177)	(0.214)
affected	0.007	-0.311	0.015	0.187	0.115
	(0.692)	(0.378)	(0.183)	(0.167)	(0.202)
deaths	-1.326*	-0.577	-0.054	-0.396**	-0.299
	(0.772)	(0.422)	(0.204)	(0.186)	(0.225)
Constant	6.094***	2.727***	1.236***	1.004***	1.128***
	(0.502)	(0.274)	(0.132)	(0.121)	(0.146)
Observations	549	549	549	549	549
R^2	0.011	0.017	0.002	0.010	0.008
Adjusted R^2	0.004	0.010	-0.005	0.003	0.001
Residual Std. Error (df = 544)	7.145	3.901	1.886	1.724	2.084
F Statistic (df = 4; 544)	1.497	2.343*	0.309	1.430	1.148
Note:			*p<0.1; **p<0.05; ***p<0.01		

Tables 27-28 contain multiple regression models of each type of adaptation action with respect to adaptive capacity control variables

Table 27: Adaptation action types with respect to adaptive capacity control variables

	Dependent variable:				
	All	Specific	Expansive	General	Other
	(1)	(2)	(3)	(4)	(5)
political stability	0.737***	0.359**	0.141**	0.145**	0.092
	(0.265)	(0.143)	(0.071)	(0.064)	(0.079)
meritocracy	-0.056	0.326	-0.276	-0.166	0.060
	(0.685)	(0.371)	(0.183)	(0.166)	(0.203)
stakeholder diversity	-0.469	-0.508**	0.202*	-0.138	-0.025
	(0.409)	(0.221)	(0.109)	(0.099)	(0.122)
local gov. power	0.150	0.123	0.010	0.034	-0.017
	(0.249)	(0.135)	(0.067)	(0.061)	(0.074)
Constant	1.553	0.125	0.309	0.240	0.880
	(2.058)	(1.113)	(0.549)	(0.500)	(0.611)
Observations	531	531	531	531	531
R^2	0.054	0.077	0.024	0.018	0.015
Adjusted R^2	0.047	0.070	0.016	0.011	0.007
Residual Std. Error (df = 526)	7.065	3.823	1.885	1.717	2.100
F Statistic (df = 4; 526)	7.551***	11.034***	3.203**	2.477**	1.936
Note:			*p<0.1; **p<0.05; ***p<0.01		

Table 28: Adaptation actions with respect to all control variables (no baselines, no time lag)

	Dependent variable:				
	All	Specific	Expansive	General	Other
	(1)	(2)	(3)	(4)	(5)
city population	0.436^{***}	0.208^{***}	0.090^{***}	0.071^{**}	0.068^{*}
	(0.116)	(0.063)	(0.031)	(0.028)	(0.035)
political stability	0.855^{***}	0.415^{***}	0.166^{**}	0.165^{**}	0.109
	(0.265)	(0.144)	(0.071)	(0.065)	(0.080)
meritocracy	-0.173	0.283	-0.311^{*}	-0.187	0.042
	(0.689)	(0.374)	(0.185)	(0.169)	(0.207)
stakeholder diversity	-0.674	-0.608^{***}	0.161	-0.176^{*}	-0.052
	(0.413)	(0.224)	(0.111)	(0.101)	(0.124)
local gov. power	0.368	0.225	0.056	0.069	0.018
	(0.254)	(0.138)	(0.068)	(0.062)	(0.076)
Constant	-1.159	-1.174	-0.249	-0.189	0.453
	(2.170)	(1.179)	(0.582)	(0.531)	(0.651)
Observations	525	525	525	525	525
R^2	0.077	0.095	0.039	0.030	0.021
Adjusted R^2	0.068	0.086	0.029	0.020	0.011
Residual Std. Error (df = 519)	7.010	3.807	1.879	1.716	2.104
F Statistic (df = 5; 519)	8.660^{***}	10.842^{***}	4.162^{***}	3.187^{***}	2.173^{*}
Note:			*p<0.1; **p<0.05; ***p<0.01		

Tables 29-31 contain raw, normalized, and baseline multiple regression models of each type of adaptation action with respect to frequency, severity, and relevant control variables.

Table 29: Adaptation action types with respect to frequency, severity, and controls

	Dependent variable:				
	All	Specific	Expansive	General	Other
	(1)	(2)	(3)	(4)	(5)
disasters	-0.838*	-0.158	-0.519***	-0.026	-0.135
	(0.436)	(0.238)	(0.107)	(0.100)	(0.140)
damages	0.556*	0.174	0.164**	0.111	0.108
	(0.306)	(0.167)	(0.075)	(0.070)	(0.098)
affected	-0.759*	-0.265	-0.169	-0.063	-0.262*
	(0.455)	(0.248)	(0.112)	(0.104)	(0.146)
deaths	-0.404	-0.195	-0.094	-0.059	-0.057
	(0.351)	(0.192)	(0.086)	(0.080)	(0.113)
time lag	-1.242**	-0.698**	-0.415***	-0.049	-0.079
	(0.558)	(0.304)	(0.137)	(0.127)	(0.179)
city population	0.554***	0.251***	0.132***	0.089***	0.082*
	(0.141)	(0.077)	(0.035)	(0.032)	(0.045)
political stability	0.794**	0.370**	0.195**	0.108	0.121
	(0.332)	(0.181)	(0.081)	(0.076)	(0.107)
meritocracy	0.368	0.378	-0.033	-0.052	0.075
	(0.881)	(0.481)	(0.216)	(0.201)	(0.283)
stakeholder diversity	-1.162*	-0.653*	-0.426***	0.039	-0.123
	(0.654)	(0.357)	(0.161)	(0.149)	(0.210)
local gov. power	0.603*	0.321*	0.224***	0.013	0.046
	(0.323)	(0.176)	(0.079)	(0.074)	(0.104)
Constant	1.547	0.583	0.111	0.297	0.555
	(3.033)	(1.654)	(0.745)	(0.693)	(0.975)
Observations	430	430	430	430	430
R^2	0.108	0.107	0.117	0.045	0.031
Adjusted R^2	0.087	0.085	0.096	0.022	0.008
Residual Std. Error (df = 419)	6.975	3.805	1.713	1.593	2.242
F Statistic (df = 10; 419)	5.078***	5.009***	5.541***	1.974**	1.354
Note:			*p<0.1; **p<0.05; ***p<0.01		

Table 30: Adaptation action types with respect to normalized frequency, severity, and controls

	Dependent variable:				
	All	Specific	Expansive	General	Other
	(1)	(2)	(3)	(4)	(5)
disasters	1.299	1.141	-0.899*	0.520	0.537
	(2.044)	(1.110)	(0.511)	(0.465)	(0.654)
damages	-0.235	-0.116	0.028	-0.011	-0.135
	(0.452)	(0.246)	(0.113)	(0.103)	(0.145)
affected	-0.273	-0.113	-0.067	-0.033	-0.059
	(0.260)	(0.141)	(0.065)	(0.059)	(0.083)
deaths	-0.461	-0.176	-0.119*	-0.045	-0.122
	(0.280)	(0.152)	(0.070)	(0.064)	(0.090)
time lag	-1.193**	-0.689**	-0.374***	-0.052	-0.078
	(0.567)	(0.308)	(0.142)	(0.129)	(0.182)
city population	0.520***	0.240***	0.110***	0.090***	0.079*
	(0.142)	(0.077)	(0.035)	(0.032)	(0.045)
political stability	0.834**	0.377**	0.203**	0.123*	0.131
	(0.324)	(0.176)	(0.081)	(0.074)	(0.104)
meritocracy	-0.286	0.255	-0.403*	-0.072	-0.066
	(0.871)	(0.473)	(0.218)	(0.198)	(0.279)
stakeholder diversity	-0.984*	-0.755**	-0.022	-0.065	-0.142
	(0.540)	(0.293)	(0.135)	(0.123)	(0.173)
local gov. power	0.594*	0.358**	0.132*	0.043	0.060
	(0.320)	(0.174)	(0.080)	(0.073)	(0.103)
Constant	1.155	0.153	0.550	0.036	0.417
	(3.032)	(1.646)	(0.759)	(0.690)	(0.971)
Observations	430	430	430	430	430
R^2	0.101	0.109	0.076	0.044	0.031
Adjusted R^2	0.080	0.087	0.054	0.021	0.008
Residual Std. Error (df = 419)	7.003	3.801	1.752	1.594	2.241
F Statistic (df = 10; 419)	4.715***	5.108***	3.444***	1.910**	1.360
Note:					*p<0.1; **p<0.05; ***p<0.01

Table 31: Adaptation action types with respect to baseline frequency, severity, and controls

	Dependent variable:				
	All	Specific	Expansive	General	Other
	(1)	(2)	(3)	(4)	(5)
disasters	0.096	0.248	-0.335*	0.037	0.145
	(0.654)	(0.356)	(0.175)	(0.161)	(0.198)
damages	1.358*	0.602	0.267	0.164	0.326
	(0.763)	(0.415)	(0.204)	(0.188)	(0.231)
affected	-0.116	-0.255	-0.109	0.158	0.090
	(0.705)	(0.383)	(0.189)	(0.174)	(0.213)
deaths	-0.365	0.337	-0.200	-0.355*	-0.147
	(0.870)	(0.473)	(0.233)	(0.214)	(0.263)
time lag	-1.181***	-0.601***	-0.280***	-0.131	-0.169
	(0.385)	(0.209)	(0.103)	(0.095)	(0.116)
city population	0.391***	0.188***	0.077**	0.065**	0.062*
	(0.117)	(0.063)	(0.031)	(0.029)	(0.035)
political stability	0.992***	0.441***	0.229***	0.194***	0.128
	(0.277)	(0.151)	(0.074)	(0.068)	(0.084)
meritocracy	-0.351	0.312	-0.405**	-0.270	0.012
	(0.737)	(0.401)	(0.197)	(0.181)	(0.223)
stakeholder diversity	-0.526	-0.610***	0.217*	-0.116	-0.016
	(0.430)	(0.234)	(0.115)	(0.106)	(0.130)
local gov. power	0.301	0.219	0.031	0.049	0.001
	(0.258)	(0.141)	(0.069)	(0.064)	(0.078)
Constant	2.420	0.450	0.796	0.271	0.904
	(2.492)	(1.355)	(0.667)	(0.613)	(0.753)
Observations	525	525	525	525	525
R^2	0.098	0.114	0.063	0.042	0.031
Adjusted R^2	0.081	0.097	0.045	0.023	0.012
Residual Std. Error (df = 514)	6.961	3.784	1.864	1.713	2.103
F Statistic (df = 10; 514)	5.612***	6.616***	3.470***	2.253**	1.625*
Note:			*p<0.1; **p<0.05; ***p<0.01		

Table 32: Adaptation action types with respect to frequency, severity, controls, and a frequency-severity interaction term

	Dependent variable:				
	All	Specific	Expansive	General	Other
	(1)	(2)	(3)	(4)	(5)
disasters	-1.281**	-0.479	-0.479***	-0.028	-0.295
	(0.641)	(0.358)	(0.158)	(0.147)	(0.199)
damages	0.074	-0.060	0.126	0.077	-0.069
	(0.349)	(0.195)	(0.086)	(0.080)	(0.109)
time lag	-1.038**	-0.569*	-0.261**	-0.143	-0.065
	(0.526)	(0.294)	(0.130)	(0.120)	(0.164)
city population	0.442***	0.195***	0.095***	0.082***	0.071*
	(0.133)	(0.074)	(0.033)	(0.030)	(0.041)
political stability	0.884***	0.425**	0.190**	0.150**	0.119
	(0.322)	(0.180)	(0.079)	(0.074)	(0.100)
meritocracy	0.381	0.432	-0.049	-0.113	0.112
	(0.847)	(0.474)	(0.209)	(0.194)	(0.264)
stakeholder diversity	-0.864	-0.566*	-0.162	-0.078	-0.058
	(0.601)	(0.336)	(0.148)	(0.137)	(0.187)
local gov. power	0.446	0.252	0.128	0.035	0.030
	(0.316)	(0.177)	(0.078)	(0.072)	(0.098)
disasters*damages	0.281	0.156	0.023	-0.004	0.107
	(0.238)	(0.133)	(0.059)	(0.055)	(0.074)
Constant	1.780	0.600	0.146	0.381	0.653
	(3.007)	(1.682)	(0.742)	(0.688)	(0.936)
Observations	454	454	454	454	454
R^2	0.105	0.107	0.093	0.044	0.030
Adjusted R^2	0.087	0.089	0.074	0.024	0.011
Residual Std. Error (df = 444)	7.061	3.950	1.743	1.616	2.198
F Statistic (df = 9; 444)	5.781***	5.926***	5.029***	2.247**	1.547
Note:			*p<0.1; **p<0.05; ***p<0.01		

Tables 33-36 contain multiple regression models of each type of adaptation action with respect to disaster frequency and severity measures, individually, and all control variables.

Table 33: Adaptation action types with respect to disaster frequency and controls

	Dependent variable:				
	All	Specific	Expansive	General	Other
	(1)	(2)	(3)	(4)	(5)
disasters	-0.631*	-0.054	-0.451***	-0.092	-0.034
	(0.374)	(0.204)	(0.099)	(0.092)	(0.113)
time lag	-1.142***	-0.543***	-0.270***	-0.156*	-0.172
	(0.375)	(0.205)	(0.099)	(0.093)	(0.114)
city population	0.412***	0.191***	0.091***	0.067**	0.063*
	(0.116)	(0.064)	(0.031)	(0.029)	(0.035)
political stability	1.014***	0.449***	0.255***	0.188***	0.123
	(0.271)	(0.148)	(0.072)	(0.067)	(0.082)
meritocracy	-0.037	0.261	-0.172	-0.166	0.041
	(0.695)	(0.379)	(0.184)	(0.171)	(0.211)
stakeholder diversity	-1.154**	-0.628**	-0.207	-0.247*	-0.072
	(0.516)	(0.282)	(0.136)	(0.127)	(0.156)
local gov. power	0.477*	0.237*	0.131*	0.085	0.024
	(0.259)	(0.141)	(0.068)	(0.064)	(0.078)
Constant	1.884	0.474	0.226	0.222	0.962
	(2.463)	(1.344)	(0.650)	(0.607)	(0.746)
Observations	525	525	525	525	525
R^2	0.098	0.107	0.088	0.037	0.025
Adjusted R^2	0.086	0.095	0.075	0.024	0.012
Residual Std. Error (df = 517)	6.944	3.789	1.834	1.713	2.103
F Statistic (df = 7; 517)	8.003***	8.831***	7.091***	2.826***	1.891*
Note:					*p<0.1; **p<0.05; ***p<0.01

Table 34: Adaptation action types with respect to disaster damages and controls.

	Dependent variable:				
	All	Specific	Expansive	General	Other
	(1)	(2)	(3)	(4)	(5)
damages	0.300	0.078	0.123*	0.072	0.028
	(0.266)	(0.148)	(0.067)	(0.061)	(0.083)
time lag	-1.216**	-0.651**	-0.303**	-0.144	-0.118
	(0.515)	(0.287)	(0.129)	(0.117)	(0.160)
city population	0.424***	0.187**	0.089***	0.082***	0.066
	(0.133)	(0.074)	(0.033)	(0.030)	(0.041)
political stability	0.819***	0.421**	0.132*	0.144**	0.121
	(0.316)	(0.177)	(0.079)	(0.072)	(0.098)
meritocracy	-0.009	0.317	-0.246	-0.128	0.048
	(0.827)	(0.461)	(0.207)	(0.188)	(0.257)
stakeholder diversity	-0.298	-0.433	0.178	-0.050	0.007
	(0.511)	(0.285)	(0.128)	(0.116)	(0.159)
local gov. power	0.329	0.219	0.067	0.031	0.012
	(0.311)	(0.174)	(0.078)	(0.071)	(0.097)
Constant	2.427	0.767	0.511	0.410	0.739
	(2.995)	(1.671)	(0.751)	(0.682)	(0.930)
Observations	454	454	454	454	454
R^2	0.096	0.104	0.054	0.043	0.025
Adjusted R^2	0.082	0.089	0.040	0.028	0.010
Residual Std. Error (df = 446)	7.080	3.949	1.775	1.612	2.199
F Statistic (df = 7; 446)	6.779***	7.361***	3.666***	2.878***	1.644
Note:					*p<0.1; **p<0.05; ***p<0.01

Table 35: Adaptation action types with respect to affected population and controls.

	Dependent variable:				
	All	Specific	Expansive	General	Other
	(1)	(2)	(3)	(4)	(5)
affected	-0.570	-0.242	-0.025	-0.061	-0.241*
	(0.407)	(0.215)	(0.109)	(0.097)	(0.126)
time lag	-1.289***	-0.528**	-0.474***	-0.138	-0.150
	(0.433)	(0.229)	(0.116)	(0.103)	(0.135)
city population	0.422***	0.203***	0.084***	0.072**	0.063*
	(0.118)	(0.062)	(0.031)	(0.028)	(0.037)
political stability	0.758***	0.328**	0.171**	0.163**	0.096
	(0.279)	(0.147)	(0.074)	(0.066)	(0.087)
meritocracy	-0.007	0.492	-0.407**	-0.172	0.081
	(0.732)	(0.387)	(0.195)	(0.175)	(0.228)
stakeholder diversity	-0.745	-0.543**	-0.033	-0.106	-0.064
	(0.460)	(0.243)	(0.123)	(0.110)	(0.143)
local gov. power	0.470*	0.252*	0.141**	0.057	0.020
	(0.265)	(0.140)	(0.071)	(0.063)	(0.083)
Constant	2.734	0.581	0.833	0.289	1.030
	(2.544)	(1.345)	(0.680)	(0.607)	(0.791)
Observations	484	484	484	484	484
R^2	0.097	0.112	0.067	0.037	0.030
Adjusted R^2	0.083	0.099	0.053	0.023	0.016
Residual Std. Error (df = 476)	6.935	3.666	1.853	1.655	2.157
F Statistic (df = 7; 476)	7.264***	8.561***	4.868***	2.621**	2.099**
Note:			*p<0.1; **p<0.05; ***p<0.01		

Table 36: Adaptation action types with respect to deaths and controls.

	Dependent variable:				
	All	Specific	Expansive	General	Other
	(1)	(2)	(3)	(4)	(5)
deaths	-0.127	-0.044	-0.013	-0.032	-0.038
	(0.319)	(0.177)	(0.085)	(0.073)	(0.098)
time lag	-0.983**	-0.493**	-0.247**	-0.119	-0.124
	(0.431)	(0.239)	(0.115)	(0.098)	(0.133)
city population	0.458***	0.211***	0.094***	0.079***	0.074**
	(0.121)	(0.067)	(0.032)	(0.028)	(0.037)
political stability	0.986***	0.479***	0.189***	0.196***	0.122
	(0.271)	(0.151)	(0.072)	(0.062)	(0.083)
meritocracy	-0.555	0.069	-0.364*	-0.256	-0.003
	(0.706)	(0.392)	(0.188)	(0.161)	(0.217)
stakeholder diversity	-0.705	-0.613**	0.150	-0.181*	-0.061
	(0.430)	(0.239)	(0.115)	(0.098)	(0.132)
local gov. power	0.422	0.244*	0.073	0.077	0.027
	(0.265)	(0.147)	(0.071)	(0.060)	(0.082)
Constant	1.367	0.256	0.324	-0.004	0.790
	(2.568)	(1.426)	(0.684)	(0.585)	(0.790)
Observations	490	490	490	490	490
R^2	0.088	0.095	0.051	0.044	0.022
Adjusted R^2	0.075	0.082	0.037	0.030	0.008
Residual Std. Error (df = 482)	6.962	3.865	1.854	1.585	2.142
F Statistic (df = 7; 482)	6.663***	7.255***	3.707***	3.187***	1.544

Note: *p<0.1; **p<0.05; ***p<0.01

Tables 37-44 contain multiple regression models of each type of adaptation action with respect to normalized disaster frequency and severity measures, individually, and all control variables.

Table 37: Adaptation action types with respect to normalized disaster frequency and controls.

	Dependent variable:				
	All	Specific	Expansive	General	Other
	(1)	(2)	(3)	(4)	(5)
disasters	0.806	1.106	-0.508	-0.064	0.271
	(1.608)	(0.874)	(0.431)	(0.396)	(0.486)
time lag	-1.190***	-0.592***	-0.252**	-0.160*	-0.186
	(0.385)	(0.209)	(0.103)	(0.095)	(0.116)
city population	0.394***	0.188***	0.080**	0.065**	0.061*
	(0.116)	(0.063)	(0.031)	(0.029)	(0.035)
political stability	0.899***	0.406***	0.203***	0.180***	0.111
	(0.271)	(0.147)	(0.073)	(0.067)	(0.082)
meritocracy	-0.276	0.276	-0.369**	-0.216	0.033
	(0.692)	(0.376)	(0.186)	(0.170)	(0.209)
stakeholder diversity	-0.683	-0.687***	0.230*	-0.157	-0.068
	(0.440)	(0.239)	(0.118)	(0.108)	(0.133)
local gov. power	0.366	0.259*	0.027	0.058	0.023
	(0.264)	(0.143)	(0.071)	(0.065)	(0.080)
Constant	2.566	0.382	0.792	0.406	0.987
	(2.537)	(1.379)	(0.680)	(0.625)	(0.766)
Observations	524	524	524	524	524
R^2	0.093	0.109	0.054	0.035	0.025
Adjusted R^2	0.081	0.097	0.041	0.022	0.012
Residual Std. Error (df = 516)	6.965	3.787	1.868	1.715	2.105
F Statistic (df = 7; 516)	7.598***	9.052***	4.223***	2.702***	1.927*
Note:	*p<0.1; **p<0.05; ***p<0.01				

Table 38: Adaptation action types with respect to normalized disaster damages and controls.

	Dependent variable:				
	All	Specific	Expansive	General	Other
	(1)	(2)	(3)	(4)	(5)
damages	-0.246	-0.139	0.097	-0.034	-0.171
	(0.391)	(0.218)	(0.098)	(0.089)	(0.121)
time lag	-1.070**	-0.598**	-0.283**	-0.114	-0.075
	(0.514)	(0.287)	(0.129)	(0.117)	(0.159)
city population	0.454***	0.199***	0.090***	0.088***	0.077*
	(0.134)	(0.074)	(0.034)	(0.030)	(0.041)
political stability	0.882***	0.434**	0.167**	0.160**	0.120
	(0.310)	(0.173)	(0.078)	(0.071)	(0.096)
meritocracy	-0.078	0.295	-0.263	-0.143	0.033
	(0.826)	(0.460)	(0.208)	(0.188)	(0.256)
stakeholder diversity	-0.536	-0.504*	0.105	-0.103	-0.033
	(0.479)	(0.267)	(0.120)	(0.109)	(0.148)
local gov. power	0.388	0.237	0.085	0.044	0.022
	(0.309)	(0.172)	(0.078)	(0.070)	(0.096)
Constant	1.863	0.617	0.288	0.277	0.682
	(2.957)	(1.648)	(0.743)	(0.674)	(0.916)
Observations	454	454	454	454	454
R^2	0.094	0.104	0.049	0.041	0.029
Adjusted R^2	0.080	0.090	0.034	0.025	0.014
Residual Std. Error (df = 446)	7.087	3.948	1.780	1.614	2.194
F Statistic (df = 7; 446)	6.641***	7.381***	3.302***	2.692***	1.919*
Note:	*p<0.1; **p<0.05; ***p<0.01				

Table 39: Adaptation action types with respect to normalized affected population and controls.

	Dependent variable:				
	All	Specific	Expansive	General	Other
	(1)	(2)	(3)	(4)	(5)
affected	-0.139	-0.073	-0.026	-0.005	-0.034
	(0.137)	(0.072)	(0.037)	(0.033)	(0.043)
time lag	-1.265***	-0.510**	-0.462***	-0.140	-0.152
	(0.438)	(0.231)	(0.117)	(0.104)	(0.137)
city population	0.409***	0.197***	0.083***	0.070**	0.058
	(0.118)	(0.062)	(0.031)	(0.028)	(0.037)
political stability	0.782***	0.338**	0.171**	0.166**	0.107
	(0.278)	(0.147)	(0.074)	(0.066)	(0.087)
meritocracy	-0.101	0.451	-0.413**	-0.181	0.043
	(0.730)	(0.386)	(0.195)	(0.174)	(0.228)
stakeholder diversity	-0.687	-0.519**	-0.031	-0.099	-0.039
	(0.459)	(0.242)	(0.122)	(0.109)	(0.143)
local gov. power	0.464*	0.250*	0.142**	0.056	0.016
	(0.266)	(0.140)	(0.071)	(0.063)	(0.083)
Constant	2.559	0.483	0.792	0.287	0.997
	(2.557)	(1.351)	(0.682)	(0.610)	(0.797)
Observations	484	484	484	484	484
R^2	0.095	0.111	0.068	0.036	0.024
Adjusted R^2	0.081	0.098	0.054	0.022	0.009
Residual Std. Error (df = 476)	6.942	3.667	1.852	1.656	2.164
F Statistic (df = 7; 476)	7.118***	8.522***	4.940***	2.566**	1.660

Note: *p<0.1; **p<0.05; ***p<0.01

Table 40: Adaptation action types with respect to normalized deaths and controls.

	Dependent variable:				
	All	Specific	Expansive	General	Other
	(1)	(2)	(3)	(4)	(5)
deaths	-0.469*	-0.166	-0.135*	-0.050	-0.118
	(0.263)	(0.146)	(0.070)	(0.060)	(0.081)
time lag	-0.943**	-0.479**	-0.236**	-0.114	-0.114
	(0.430)	(0.239)	(0.114)	(0.098)	(0.132)
city population	0.433***	0.202***	0.087***	0.076***	0.068*
	(0.121)	(0.067)	(0.032)	(0.028)	(0.037)
political stability	1.003***	0.485***	0.194***	0.197***	0.126
	(0.271)	(0.151)	(0.072)	(0.062)	(0.083)
meritocracy	-0.666	0.029	-0.401**	-0.264	-0.030
	(0.705)	(0.392)	(0.188)	(0.161)	(0.217)
stakeholder diversity	-0.772*	-0.637***	0.129	-0.187*	-0.078
	(0.431)	(0.240)	(0.115)	(0.098)	(0.133)
local gov. power	0.433	0.248*	0.078	0.077	0.030
	(0.264)	(0.147)	(0.070)	(0.060)	(0.081)
Constant	1.398	0.268	0.336	-0.003	0.797
	(2.560)	(1.424)	(0.681)	(0.584)	(0.789)
Observations	490	490	490	490	490
R^2	0.094	0.098	0.058	0.045	0.026
Adjusted R^2	0.081	0.085	0.045	0.031	0.012
Residual Std. Error (df = 482)	6.941	3.861	1.847	1.584	2.138
F Statistic (df = 7; 482)	7.134***	7.449***	4.258***	3.259***	1.832*
Note:			*p<0.1; **p<0.05; ***p<0.01		

Table 41: Adaptation action types with respect to baseline disaster frequency and controls.

	Dependent variable:				
	All	Specific	Expansive	General	Other
	(1)	(2)	(3)	(4)	(5)
disasters	0.118	0.323	-0.364**	0.004	0.154
	(0.634)	(0.345)	(0.169)	(0.156)	(0.191)
time lag	-1.125***	-0.532***	-0.272***	-0.154*	-0.166
	(0.377)	(0.205)	(0.101)	(0.093)	(0.114)
city population	0.397***	0.190***	0.079**	0.065**	0.062*
	(0.116)	(0.063)	(0.031)	(0.029)	(0.035)
political stability	0.901***	0.421***	0.200***	0.172***	0.108
	(0.267)	(0.145)	(0.071)	(0.066)	(0.081)
meritocracy	-0.257	0.244	-0.333*	-0.199	0.030
	(0.684)	(0.372)	(0.183)	(0.169)	(0.207)
stakeholder diversity	-0.624	-0.588***	0.179	-0.169*	-0.046
	(0.410)	(0.223)	(0.110)	(0.101)	(0.124)
local gov. power	0.375	0.233*	0.052	0.070	0.021
	(0.252)	(0.137)	(0.067)	(0.062)	(0.076)
Constant	2.310	0.384	0.712	0.290	0.924
	(2.467)	(1.341)	(0.660)	(0.607)	(0.745)
Observations	525	525	525	525	525
R^2	0.093	0.108	0.059	0.035	0.026
Adjusted R^2	0.081	0.096	0.046	0.022	0.013
Residual Std. Error (df = 517)	6.963	3.786	1.862	1.714	2.102
F Statistic (df = 7; 517)	7.561***	8.961***	4.648***	2.677***	1.973*
Note:					*p<0.1; **p<0.05; ***p<0.01

Table 42: Adaptation action types with respect to baseline disaster damages and controls.

	Dependent variable:				
	actions	specific	expansive	general	other
	(1)	(2)	(3)	(4)	(5)
damages	1.254*	0.654*	0.162	0.115	0.323
	(0.722)	(0.393)	(0.194)	(0.178)	(0.218)
time lag	-1.194***	-0.576***	-0.269***	-0.160*	-0.188*
	(0.377)	(0.205)	(0.101)	(0.093)	(0.114)
city population	0.394***	0.188***	0.080**	0.065**	0.061*
	(0.116)	(0.063)	(0.031)	(0.029)	(0.035)
political stability	0.962***	0.468***	0.186***	0.177***	0.131
	(0.265)	(0.144)	(0.071)	(0.065)	(0.080)
meritocracy	-0.224	0.260	-0.326*	-0.196	0.038
	(0.683)	(0.372)	(0.184)	(0.169)	(0.206)
stakeholder diversity	-0.580	-0.561**	0.178	-0.165	-0.033
	(0.410)	(0.223)	(0.110)	(0.101)	(0.124)
local gov. power	0.322	0.201	0.050	0.065	0.005
	(0.253)	(0.138)	(0.068)	(0.063)	(0.077)
Constant	2.316	0.493	0.559	0.288	0.976
	(2.446)	(1.332)	(0.659)	(0.604)	(0.740)
Observations	525	525	525	525	525
R^2	0.098	0.111	0.052	0.036	0.029
Adjusted R^2	0.086	0.099	0.039	0.023	0.016
Residual Std. Error (df = 517)	6.943	3.779	1.869	1.714	2.099
F Statistic (df = 7; 517)	8.030***	9.262***	4.058***	2.738***	2.199**
Note:					*p<0.1; **p<0.05; ***p<0.01

Table 43: Adaptation action types with respect to baseline affected population and controls.

	Dependent variable:				
	All	Specific	Expansive	General	Other
	(1)	(2)	(3)	(4)	(5)
affected	0.105	-0.062	-0.132	0.147	0.153
	(0.684)	(0.372)	(0.184)	(0.168)	(0.207)
time lag	-1.123***	-0.546***	-0.269***	-0.146	-0.162
	(0.378)	(0.206)	(0.102)	(0.093)	(0.114)
city population	0.397***	0.189***	0.079**	0.066**	0.063*
	(0.117)	(0.063)	(0.031)	(0.029)	(0.035)
political stability	0.903***	0.443***	0.185***	0.165**	0.110
	(0.266)	(0.145)	(0.072)	(0.066)	(0.080)
meritocracy	-0.238	0.231	-0.355*	-0.171	0.058
	(0.696)	(0.379)	(0.187)	(0.171)	(0.210)
stakeholder diversity	-0.624	-0.582***	0.175	-0.171*	-0.046
	(0.410)	(0.223)	(0.110)	(0.101)	(0.124)
local gov. power	0.375	0.227*	0.055	0.072	0.020
	(0.252)	(0.137)	(0.068)	(0.062)	(0.076)
Constant	2.302	0.548	0.634	0.214	0.906
	(2.480)	(1.350)	(0.666)	(0.610)	(0.749)
Observations	525	525	525	525	525
R^2	0.093	0.107	0.052	0.036	0.026
Adjusted R^2	0.081	0.095	0.039	0.023	0.013
Residual Std. Error (df = 517)	6.963	3.789	1.870	1.713	2.102
F Statistic (df = 7; 517)	7.559***	8.824***	4.032***	2.790***	1.958*
Note:	*p<0.1; **p<0.05; ***p<0.01				

Table 44: Adaptation action types with respect to baseline deaths and controls.

	Dependent variable:				
	All	Specific	Expansive	General	Other
	(1)	(2)	(3)	(4)	(5)
deaths	0.057	0.547	-0.238	-0.265	0.013
	(0.814)	(0.442)	(0.218)	(0.200)	(0.246)
time lag	-1.132***	-0.575***	-0.247**	-0.139	-0.172
	(0.379)	(0.206)	(0.102)	(0.093)	(0.115)
city population	0.397***	0.192***	0.079**	0.064**	0.062*
	(0.116)	(0.063)	(0.031)	(0.029)	(0.035)
political stability	0.904***	0.402***	0.195***	0.191***	0.116
	(0.270)	(0.147)	(0.072)	(0.066)	(0.082)
meritocracy	-0.241	0.405	-0.401**	-0.277	0.033
	(0.726)	(0.394)	(0.195)	(0.178)	(0.219)
stakeholder diversity	-0.630	-0.659***	0.206*	-0.132	-0.045
	(0.426)	(0.231)	(0.114)	(0.105)	(0.129)
local gov. power	0.376	0.253*	0.046	0.058	0.019
	(0.255)	(0.139)	(0.068)	(0.063)	(0.077)
Constant	2.344	0.387	0.620	0.354	0.984
	(2.461)	(1.337)	(0.660)	(0.605)	(0.743)
Observations	525	525	525	525	525
R^2	0.093	0.109	0.053	0.038	0.025
Adjusted R^2	0.081	0.097	0.040	0.025	0.012
Residual Std. Error (df = 517)	6.963	3.783	1.868	1.711	2.104
F Statistic (df = 7; 517)	7.556***	9.065***	4.132***	2.936***	1.878*
Note:					*p<0.1; **p<0.05; ***p<0.01

Tables 45-56 contain multiple regression models of each type of adaptation action with respect to disaster frequency and severity measures, individually, and all control variables.

Table 45: All adaptation actions with respect to disaster frequency, controls, and interaction terms.

	Dependent variable:				
	All actions				
	(1)	(2)	(3)	(4)	(5)
disasters	0.466	0.133	-0.705	0.476	-2.095
	(0.289)	(1.637)	(1.691)	(0.679)	(2.749)
time lag	-0.915**	-1.127***	-0.995***	-0.930**	-0.974**
	(0.378)	(0.375)	(0.377)	(0.380)	(0.382)
city population	0.113	0.355***	0.304***	0.240**	0.306***
	(0.165)	(0.113)	(0.113)	(0.114)	(0.117)
disasters*city pop.	0.167				
	(0.170)				
political stability		0.868***			
		(0.187)			
disasters*political stability		-0.038			
		(0.211)			
meritocracy			1.686***		
			(0.525)		
disasters*meritocracy			0.337		
			(0.851)		
stakeholder diversity				0.507	
				(0.547)	
disasters*stakeholder diversity				0.477	
				(0.974)	
local gov. power					0.394
					(0.264)
disasters*local gov. power					0.289
					(0.298)
Constant	8.937***	5.115***	7.704***	7.929***	5.411*
	(1.371)	(1.761)	(1.616)	(1.469)	(2.777)
Observations	543	532	540	540	533
R^2	0.030	0.088	0.064	0.034	0.040
Adjusted R^2	0.023	0.079	0.055	0.025	0.031
Residual Std. Error	7.098 (df = 538)	6.932 (df = 526)	6.992 (df = 534)	7.103 (df = 534)	7.116 (df = 527)
F Statistic	4.166*** (df = 4; 538)	10.098*** (df = 5; 526)	7.267*** (df = 5; 534)	3.725*** (df = 5; 534)	4.423*** (df = 5; 527)
Note:					*p<0.1; **p<0.05; ***p<0.01

Table 46: All adaptation actions with respect to disaster damages, controls, and interaction terms.

	Dependent variable:				
	All actions				
	(1)	(2)	(3)	(4)	(5)
damages	0.685**	-1.217*	-0.320	0.741	0.716
	(0.272)	(0.625)	(0.384)	(0.491)	(2.222)
time lag	-1.279**	-1.088**	-0.996**	-1.299**	-1.362***
	(0.516)	(0.503)	(0.505)	(0.527)	(0.523)
city population	0.255	0.409***	0.370***	0.268**	0.323**
	(0.166)	(0.127)	(0.127)	(0.129)	(0.133)
damages*city pop.	0.010				
	(0.091)				
political stability		0.480**			
		(0.192)			
damages*political stability		0.275***			
		(0.101)			
meritocracy			0.986*		
			(0.532)		
damages*meritocracy			0.700***		
			(0.270)		
stakeholder diversity				0.546	
				(0.531)	
damages*stakeholder diversity				0.046	
				(0.416)	
local gov. power					0.529*
					(0.292)
damages*local gov. power					-0.003
					(0.238)
Constant	9.862***	6.343***	7.532***	9.153***	5.197
	(1.732)	(1.972)	(1.800)	(1.976)	(3.166)
Observations	465	458	465	465	461
R^2	0.037	0.107	0.090	0.041	0.045
Adjusted R^2	0.028	0.097	0.080	0.030	0.034
Residual Std. Error	7.214 (df = 460)	6.996 (df = 452)	7.019 (df = 459)	7.208 (df = 459)	7.220 (df = 455)
F Statistic	4.386*** (df = 4; 460)	10.794*** (df = 5; 452)	9.111*** (df = 5; 459)	3.884*** (df = 5; 459)	4.259*** (df = 5; 455)
Note:					*p<0.1; **p<0.05; ***p<0.01

Table 47: All adaptation actions with respect to affected population, controls, and interaction terms.

	Dependent variable:				
	All actions				
	(1)	(2)	(3)	(4)	(5)
affected	-0.477	-1.457	-0.995	-0.177	-0.076
	(0.619)	(1.902)	(1.119)	(0.718)	(3.970)
time lag	-1.135***	-1.126***	-0.974**	-1.023**	-1.192***
	(0.434)	(0.429)	(0.436)	(0.449)	(0.446)
city population	0.244**	0.375***	0.336***	0.240**	0.305**
	(0.116)	(0.114)	(0.114)	(0.114)	(0.118)
affected*city pop.	-0.029				
	(0.200)				
political stability		0.746***			
		(0.141)			
affected*political stability		0.184			
		(0.363)			
meritocracy			1.752***		
			(0.386)		
affected*meritocracy			0.281		
			(0.777)		
stakeholder diversity				0.145	
				(0.400)	
affected*stakeholder diversity				-0.596	
				(0.965)	
local gov. power					0.555**
					(0.232)
affected*local gov. power					-0.053
					(0.468)
Constant	10.150***	5.710***	7.481***	9.657***	5.256**
	(1.480)	(1.653)	(1.555)	(1.637)	(2.594)
Observations	502	491	499	499	492
R^2	0.030	0.090	0.072	0.030	0.041
Adjusted R^2	0.022	0.080	0.063	0.020	0.031
Residual Std. Error	7.077 (df = 497)	6.907 (df = 485)	6.941 (df = 493)	7.098 (df = 493)	7.097 (df = 486)
F Statistic	3.793*** (df = 4; 497)	9.544*** (df = 5; 485)	7.668*** (df = 5; 493)	3.022** (df = 5; 493)	4.142*** (df = 5; 486)
Note:					*p<0.1; **p<0.05; ***p<0.01

Table 48: All adaptation actions with respect to disaster deaths, controls, and interaction terms.

	Dependent variable:				
	All actions				
	(1)	(2)	(3)	(4)	(5)
deaths	-0.257	-0.308	-0.187	-1.215	-4.753
	(0.363)	(0.854)	(0.574)	(0.818)	(3.817)
time lag	-0.605	-0.917**	-0.721*	-0.572	-0.700
	(0.432)	(0.432)	(0.432)	(0.434)	(0.436)
city population	0.250*	0.399***	0.335***	0.286**	0.389***
	(0.139)	(0.117)	(0.116)	(0.118)	(0.129)
deaths*city pop.	0.019				
	(0.091)				
political stability		0.776***			
		(0.150)			
deaths*political stability		0.034			
		(0.118)			
meritocracy			1.612***		
			(0.424)		
deaths*meritocracy			0.098		
			(0.345)		
stakeholder diversity				0.007	
				(0.398)	
deaths*stakeholder diversity				0.666	
				(0.506)	
local gov. power					0.429*
					(0.256)
deaths*local gov. power					0.472
					(0.400)
Constant	8.496***	4.772***	6.713***	8.437***	4.838*
	(1.498)	(1.625)	(1.525)	(1.554)	(2.735)
Observations	505	495	503	503	498
R^2	0.016	0.079	0.054	0.020	0.030
Adjusted R^2	0.008	0.070	0.044	0.010	0.020
Residual Std. Error	7.137 (df = 500)	6.950 (df = 489)	7.013 (df = 497)	7.139 (df = 497)	7.133 (df = 492)
F Statistic	2.062* (df = 4; 500)	8.424*** (df = 5; 489)	5.655*** (df = 5; 497)	1.985* (df = 5; 497)	3.038** (df = 5; 492)
Note:					*p<0.1; **p<0.05; ***p<0.01

Table 49: Specific and expansive adaptation actions with respect to disaster frequency, controls, and interaction terms

	Dependent variable:									
	Specific actions					Expansive actions				
	(1)	(2)	(3)	(4)	(5)	(1)	(2)	(3)	(4)	(5)
disasters	0.531***	0.439	0.188	0.350	-0.898	-0.278***	-0.293	-0.690	0.132	-0.102
	(0.157)	(0.892)	(0.917)	(0.368)	(1.495)	(0.075)	(0.433)	(0.447)	(0.177)	(0.719)
time.lag	-0.461**	-0.547***	-0.490**	-0.454**	-0.477**	-0.212**	-0.254**	-0.230**	-0.225**	-0.227**
	(0.205)	(0.204)	(0.204)	(0.206)	(0.208)	(0.099)	(0.099)	(0.100)	(0.099)	(0.100)
city.pop	0.002	0.163***	0.139**	0.098	0.135**	-0.003	0.075**	0.062**	0.059**	0.067**
	(0.089)	(0.061)	(0.061)	(0.062)	(0.064)	(0.043)	(0.030)	(0.030)	(0.030)	(0.031)
disasters*city.pop	0.137					0.074*				
	(0.092)					(0.044)				
political stability		0.455***					0.188***			
		(0.102)					(0.049)			
disasters*political stability		-0.027					-0.012			
		(0.115)					(0.056)			
meritocracy			1.008***					0.257*		
			(0.284)					(0.139)		
disasters*meritocracy			0.043					0.179		
			(0.461)					(0.225)		
stakeholder diversity				0.103					0.389***	
				(0.297)					(0.143)	
disasters*stakeholder diversity				0.582					-0.479*	
				(0.528)					(0.254)	
local gov. power					0.166					0.163**
					(0.144)					(0.069)
disasters*local gov. power					0.164					-0.014
					(0.162)					(0.078)
Constant	3.970***	1.872*	3.086***	3.498***	2.426	2.095***	1.232***	1.950***	1.666***	0.593
	(0.743)	(0.960)	(0.876)	(0.797)	(1.511)	(0.358)	(0.466)	(0.427)	(0.383)	(0.726)
Observations	543	532	540	540	533	543	532	540	540	533
R^2	0.051	0.098	0.084	0.052	0.055	0.041	0.076	0.054	0.049	0.050
Adjusted R^2	0.044	0.090	0.075	0.043	0.046	0.034	0.067	0.045	0.040	0.041
Residual Std. Error	3.849 (df = 538)	3.778 (df = 526)	3.790 (df = 534)	3.854 (df = 534)	3.870 (df = 527)	1.856 (df = 538)	1.834 (df = 526)	1.849 (df = 534)	1.854 (df = 534)	1.861 (df = 527)
F Statistic	7.226*** (df = 4; 538)	11.456*** (df = 5; 526)	9.748*** (df = 5; 534)	5.876*** (df = 5; 534)	6.100*** (df = 5; 527)	5.697*** (df = 4; 538)	8.608*** (df = 5; 526)	6.072*** (df = 5; 534)	5.498*** (df = 5; 534)	5.580*** (df = 5; 527)
Note:										*p<0.1; **p<0.05; ***p<0.01

Table 50: Specific and expansive adaptation actions with respect to disaster damages, controls, and interaction terms.

	Dependent variable:									
	Specific actions					Expansive actions				
	(1)	(2)	(3)	(4)	(5)	(1)	(2)	(3)	(4)	(5)
damages	0.393**	-0.561	-0.233	0.879***	0.830	0.075	0.079	0.133	-0.056	0.003
	(0.153)	(0.350)	(0.214)	(0.275)	(1.252)	(0.067)	(0.159)	(0.098)	(0.120)	(0.546)
time.lag	-0.725**	-0.611**	-0.544*	-0.601**	-0.749**	-0.282**	-0.258**	-0.269**	-0.338***	-0.315**
	(0.290)	(0.282)	(0.281)	(0.296)	(0.295)	(0.127)	(0.128)	(0.128)	(0.129)	(0.128)
city.pop	0.094	0.175**	0.154**	0.107	0.110	0.036	0.081**	0.073**	0.064**	0.089***
	(0.093)	(0.071)	(0.071)	(0.073)	(0.075)	(0.041)	(0.033)	(0.032)	(0.032)	(0.033)
damages*city.pop	-0.006					0.030				
	(0.051)					(0.022)				
political stability		0.350***					0.071			
		(0.108)					(0.049)			
damages*political stability		0.126**					0.001			
		(0.057)					(0.026)			
meritocracy			0.730**					0.108		
			(0.296)					(0.135)		
damages*meritocracy			0.412***					-0.024		
			(0.150)					(0.068)		
stakeholder diversity				0.459					0.134	
				(0.298)					(0.130)	
damages*stakeholder diversity				-0.472**					0.215**	
				(0.234)					(0.102)	
local gov. power					0.262					0.156**
					(0.165)					(0.072)
damages*local gov. power					-0.049					0.012
					(0.134)					(0.058)
Constant	5.113***	2.733**	3.540***	4.094***	2.784	1.828***	1.311***	1.624***	1.774***	0.427
	(0.975)	(1.104)	(1.003)	(1.109)	(1.784)	(0.427)	(0.503)	(0.457)	(0.482)	(0.778)
Observations	465	458	465	465	461	465	458	465	465	461
R^2	0.030	0.107	0.102	0.039	0.035	0.031	0.034	0.029	0.054	0.042
Adjusted R^2	0.021	0.097	0.092	0.028	0.024	0.023	0.023	0.019	0.043	0.031
Residual Std. Error	4.059 (df = 460)	3.917 (df = 452)	3.910 (df = 459)	4.045 (df = 459)	4.069 (df = 455)	1.777 (df = 460)	1.786 (df = 452)	1.781 (df = 459)	1.759 (df = 459)	1.774 (df = 455)
F Statistic	3.528*** (df = 4; 460)	10.841*** (df = 5; 452)	10.392*** (df = 5; 459)	3.693*** (df = 5; 459)	3.265*** (df = 5; 455)	3.725*** (df = 4; 460)	3.177*** (df = 5; 452)	2.750** (df = 5; 459)	5.200*** (df = 5; 459)	3.981*** (df = 5; 455)
Note:										*p<0.1; **p<0.05; ***p<0.01

Table 51: Specific and expansive adaptation actions with respect to affected population, controls, and interaction terms.

	Dependent variable:									
	Specific actions					Expansive actions				
	(1)	(2)	(3)	(4)	(5)	(1)	(2)	(3)	(4)	(5)
affected	-0.230	-0.861	-0.601	0.174	1.100	0.050	0.066	0.228	-0.263	-1.626
	(0.332)	(1.008)	(0.590)	(0.385)	(2.132)	(0.162)	(0.513)	(0.300)	(0.187)	(1.035)
time.lag	-0.488**	-0.456**	-0.374	-0.407*	-0.486**	-0.420***	-0.424***	-0.434***	-0.440***	-0.480***
	(0.233)	(0.227)	(0.230)	(0.240)	(0.240)	(0.114)	(0.116)	(0.117)	(0.117)	(0.116)
city.pop	0.091	0.178***	0.160***	0.090	0.123*	0.065**	0.069**	0.061**	0.062**	0.080***
	(0.062)	(0.061)	(0.060)	(0.061)	(0.063)	(0.030)	(0.031)	(0.030)	(0.030)	(0.031)
affected*city.pop	0.022					-0.045				
	(0.107)					(0.052)				
political stability		0.465***					0.061			
		(0.075)					(0.038)			
affected*political stability		0.135					-0.023			
		(0.193)					(0.098)			
meritocracy			1.219***					0.039		
			(0.203)					(0.103)		
affected*meritocracy			0.275					-0.211		
			(0.409)					(0.208)		
stakeholder diversity				-0.062					0.137	
				(0.214)					(0.104)	
affected*stakeholder diversity				-0.610					0.371	
				(0.517)					(0.252)	
local gov. power					0.270**					0.147**
					(0.124)					(0.060)
affected*local gov. power					-0.151					0.187
					(0.251)					(0.122)
Constant	4.645***	1.806**	2.774***	4.490***	2.182	2.429***	2.093***	2.436***	2.327***	1.254*
	(0.794)	(0.877)	(0.819)	(0.876)	(1.393)	(0.387)	(0.445)	(0.416)	(0.427)	(0.676)
Observations	502	491	499	499	492	502	491	499	499	492
R^2	0.017	0.099	0.092	0.020	0.026	0.040	0.045	0.040	0.047	0.059
Adjusted R^2	0.010	0.090	0.083	0.010	0.016	0.032	0.035	0.031	0.037	0.050
Residual Std. Error	3.797 (df = 497)	3.662 (df = 485)	3.658 (df = 493)	3.800 (df = 493)	3.810 (df = 486)	1.852 (df = 497)	1.861 (df = 485)	1.859 (df = 493)	1.852 (df = 493)	1.849 (df = 486)
F Statistic	2.208* (df = 4; 497)	10.696*** (df = 5; 485)	9.983*** (df = 5; 493)	1.976* (df = 5; 493)	2.621** (df = 5; 486)	5.177*** (df = 4; 497)	4.526*** (df = 5; 485)	4.140*** (df = 5; 493)	4.851*** (df = 5; 493)	6.138*** (df = 5; 486)
Note:										*p<0.1; **p<0.05; ***p<0.01

Table 52: Specific and expansive adaptation actions with respect to disaster deaths, controls, and interaction terms.

	Dependent variable:									
	Specific actions					Expansive actions				
	(1)	(2)	(3)	(4)	(5)	(1)	(2)	(3)	(4)	(5)
deaths	-0.083	0.155	0.157	-0.718	-2.166	-0.102	0.056	-0.031	0.160	0.088
	(0.203)	(0.475)	(0.318)	(0.457)	(2.139)	(0.095)	(0.229)	(0.153)	(0.213)	(0.997)
time.lag	-0.311	-0.497**	-0.422*	-0.277	-0.350	-0.173	-0.220*	-0.176	-0.193*	-0.203*
	(0.242)	(0.240)	(0.239)	(0.243)	(0.244)	(0.113)	(0.116)	(0.115)	(0.113)	(0.114)
city.pop	0.110	0.177***	0.145**	0.109*	0.153**	0.027	0.081***	0.071**	0.065**	0.088***
	(0.078)	(0.065)	(0.064)	(0.066)	(0.072)	(0.036)	(0.031)	(0.031)	(0.031)	(0.034)
deaths*city.pop	-0.015					0.049**				
	(0.051)					(0.024)				
political stability		0.494***					0.094**			
		(0.083)					(0.040)			
deaths*political stability		-0.027					-0.008			
		(0.065)					(0.032)			
meritocracy			1.188***					0.080		
			(0.234)					(0.113)		
deaths*meritocracy			-0.110					0.020		
			(0.191)					(0.092)		
stakeholder diversity				-0.213					0.303***	
				(0.222)					(0.103)	
deaths*stakeholder diversity				0.405					-0.113	
				(0.283)					(0.131)	
local gov. power					0.194					0.166**
					(0.143)					(0.067)
deaths*local gov. power					0.214					-0.011
					(0.224)					(0.104)
Constant	4.153***	1.770*	2.923***	4.363***	2.519	1.673***	1.202***	1.537***	1.309***	0.180
	(0.837)	(0.904)	(0.845)	(0.868)	(1.532)	(0.390)	(0.436)	(0.407)	(0.404)	(0.714)
Observations	505	495	503	503	498	505	495	503	503	498
R^2	0.009	0.083	0.065	0.013	0.018	0.025	0.028	0.018	0.033	0.031
Adjusted R^2	0.001	0.074	0.056	0.003	0.008	0.017	0.018	0.008	0.023	0.021
Residual Std. Error	3.990 (df = 500)	3.866 (df = 489)	3.883 (df = 497)	3.989 (df = 497)	3.997 (df = 492)	1.858 (df = 500)	1.865 (df = 489)	1.869 (df = 497)	1.855 (df = 497)	1.863 (df = 492)
F Statistic	1.174 (df = 4; 500)	8.878*** (df = 5; 489)	6.907*** (df = 5; 497)	1.346 (df = 5; 497)	1.824 (df = 5; 492)	3.150** (df = 4; 500)	2.862** (df = 5; 489)	1.801 (df = 5; 497)	3.373*** (df = 5; 497)	3.124*** (df = 5; 492)
Note:										*p<0.1; **p<0.05; ***p<0.01

Table 53: General and other adaptation actions with respect to disaster frequency, controls, and interaction terms.

	Dependent variable:									
	General actions					Other actions				
	(1)	(2)	(3)	(4)	(5)	(1)	(2)	(3)	(4)	(5)
disasters	0.073	0.240	0.087	0.018	-0.091	0.140	-0.253	-0.289	-0.024	-1.004
	(0.070)	(0.403)	(0.418)	(0.166)	(0.672)	(0.085)	(0.495)	(0.505)	(0.200)	(0.810)
time.lag	-0.110	-0.148	-0.119	-0.113	-0.124	-0.132	-0.178	-0.156	-0.138	-0.146
	(0.092)	(0.092)	(0.093)	(0.093)	(0.093)	(0.111)	(0.113)	(0.112)	(0.112)	(0.112)
city.pop	0.043	0.057^{**}	0.050^{*}	0.042	0.053^{*}	0.072	0.060^{*}	0.053	0.041	0.051
	(0.040)	(0.028)	(0.028)	(0.028)	(0.029)	(0.049)	(0.034)	(0.034)	(0.034)	(0.035)
disasters*city.pop	-0.001					-0.043				
	(0.041)					(0.050)				
political stability		0.125^{***}					0.100^{*}			
		(0.046)					(0.056)			
disasters*political stability		-0.032					0.034			
		(0.052)					(0.064)			
meritocracy			0.206					0.215		
			(0.130)					(0.157)		
disasters*meritocracy			-0.039					0.155		
			(0.210)					(0.254)		
stakeholder diversity				-0.009					0.025	
				(0.133)					(0.161)	
disasters*stakeholder diversity				0.104					0.271	
				(0.238)					(0.287)	
local gov. power					0.072					-0.007
					(0.065)					(0.078)
disasters*local gov. power					0.018					0.121
					(0.073)					(0.088)
Constant	1.303^{***}	0.721^{*}	1.122^{***}	1.284^{***}	0.691	1.569^{***}	1.289^{**}	1.547^{***}	1.481^{***}	1.700^{**}
	(0.334)	(0.434)	(0.399)	(0.359)	(0.679)	(0.404)	(0.532)	(0.482)	(0.433)	(0.819)
Observations	543	532	540	540	533	543	532	540	540	533
R^2	0.010	0.029	0.017	0.011	0.015	0.011	0.024	0.020	0.013	0.014
Adjusted R^2	0.003	0.020	0.008	0.002	0.005	0.004	0.015	0.011	0.004	0.005
Residual Std. Error	1.731 (df = 538)	1.707 (df = 526)	1.729 (df = 534)	1.734 (df = 534)	1.740 (df = 527)	2.090 (df = 538)	2.096 (df = 526)	2.087 (df = 534)	2.094 (df = 534)	2.097 (df = 527)
F Statistic	1.358 (df = 4; 538)	3.152^{***} (df = 5; 526)	1.860^{*} (df = 5; 534)	1.172 (df = 5; 534)	1.557 (df = 5; 527)	1.514 (df = 4; 538)	2.598^{**} (df = 5; 526)	2.152^{*} (df = 5; 534)	1.456 (df = 5; 534)	1.507 (df = 5; 527)
Note:										*p<0.1; **p<0.05; ***p<0.01

Table 54: General and other adaptation actions with respect to disaster damages, controls, and interaction terms.

	Dependent variable:									
	General actions					Other actions				
	(1)	(2)	(3)	(4)	(5)	(1)	(2)	(3)	(4)	(5)
damages	0.125**	-0.303**	-0.053	-0.176	0.093	0.091	-0.432**	-0.166	0.094	-0.210
	(0.061)	(0.142)	(0.088)	(0.109)	(0.500)	(0.083)	(0.195)	(0.119)	(0.150)	(0.677)
time.lag	-0.140	-0.114	-0.104	-0.223*	-0.153	-0.132	-0.106	-0.079	-0.137	-0.145
	(0.116)	(0.114)	(0.115)	(0.117)	(0.118)	(0.157)	(0.157)	(0.156)	(0.161)	(0.159)
city.pop	0.074**	0.082***	0.079***	0.054*	0.073**	0.051	0.071*	0.064	0.044	0.050
	(0.037)	(0.029)	(0.029)	(0.029)	(0.030)	(0.050)	(0.040)	(0.039)	(0.039)	(0.040)
damages*city.pop	-0.007					-0.007				
	(0.020)					(0.028)				
political stability		0.025					0.034			
		(0.044)					(0.060)			
damages*political stability		0.068***					0.080**			
		(0.023)					(0.032)			
meritocracy			0.068					0.080		
			(0.122)					(0.165)		
damages*meritocracy			0.125**					0.188**		
			(0.062)					(0.083)		
stakeholder diversity				-0.147					0.100	
				(0.118)					(0.162)	
damages*stakeholder diversity				0.299***					0.005	
				(0.093)					(0.127)	
local gov. power					0.071					0.040
					(0.066)					(0.089)
damages*local gov. power					0.002					0.032
					(0.054)					(0.072)
Constant	1.284***	0.987**	1.054**	1.763***	0.690	1.636***	1.312**	1.314**	1.522**	1.296
	(0.389)	(0.448)	(0.412)	(0.439)	(0.712)	(0.527)	(0.614)	(0.557)	(0.602)	(0.964)
Observations	465	458	465	465	461	465	458	465	465	461
R^2	0.023	0.061	0.043	0.046	0.025	0.007	0.037	0.030	0.008	0.008
Adjusted R^2	0.015	0.051	0.033	0.036	0.015	-0.002	0.026	0.020	-0.003	-0.003
Residual Std. Error	1.620 (df = 460)	1.588 (df = 452)	1.605 (df = 459)	1.602 (df = 459)	1.624 (df = 455)	2.197 (df = 460)	2.180 (df = 452)	2.173 (df = 459)	2.198 (df = 459)	2.198 (df = 455)
F Statistic	2.729** (df = 4; 460)	5.898*** (df = 5; 452)	4.140*** (df = 5; 459)	4.424*** (df = 5; 459)	2.375** (df = 5; 455)	0.788 (df = 4; 460)	3.442*** (df = 5; 452)	2.852** (df = 5; 459)	0.746 (df = 5; 459)	0.750 (df = 5; 455)
Note:										*p<0.1; **p<0.05; ***p<0.01

Table 55: General and other adaptation actions with respect to affected population, controls, and interaction terms.

	Dependent variable:									
	General actions					Other actions				
	(1)	(2)	(3)	(4)	(5)	(1)	(2)	(3)	(4)	(5)
affected	-0.097	-0.474	-0.239	-0.014	-0.102	-0.200	-0.188	-0.383	-0.075	0.552
	(0.146)	(0.452)	(0.269)	(0.170)	(0.942)	(0.187)	(0.592)	(0.345)	(0.217)	(1.205)
time.lag	-0.083	-0.099	-0.062	-0.075	-0.104	-0.143	-0.147	-0.104	-0.100	-0.122
	(0.103)	(0.102)	(0.105)	(0.106)	(0.106)	(0.132)	(0.133)	(0.134)	(0.136)	(0.135)
city.pop	0.047*	0.066**	0.061**	0.049*	0.059**	0.041	0.061*	0.055	0.039	0.043
	(0.028)	(0.027)	(0.027)	(0.027)	(0.028)	(0.035)	(0.036)	(0.035)	(0.035)	(0.036)
affected*city.pop	0.010					-0.015				
	(0.047)					(0.060)				
political stability		0.104***					0.116***			
		(0.034)					(0.044)			
affected*political stability		0.081					-0.009			
		(0.086)					(0.113)			
meritocracy			0.213**					0.281**		
			(0.093)					(0.119)		
affected*meritocracy			0.116					0.101		
			(0.187)					(0.239)		
stakeholder diversity				0.045					0.025	
				(0.094)					(0.121)	
affected*stakeholder diversity				-0.092					-0.265	
				(0.228)					(0.292)	
local gov. power					0.086					0.052
					(0.055)					(0.070)
affected*local gov. power					0.004					-0.093
					(0.111)					(0.142)
Constant	1.298***	0.696*	0.960**	1.215***	0.574	1.778***	1.116**	1.311***	1.624***	1.246
	(0.350)	(0.393)	(0.373)	(0.387)	(0.615)	(0.448)	(0.514)	(0.478)	(0.495)	(0.787)
Observations	502	491	499	499	492	502	491	499	499	492
R^2	0.010	0.036	0.024	0.011	0.015	0.013	0.028	0.026	0.014	0.014
Adjusted R^2	0.002	0.026	0.014	0.001	0.005	0.005	0.018	0.016	0.004	0.004
Residual Std. Error	1.675 (df = 497)	1.643 (df = 485)	1.667 (df = 493)	1.678 (df = 493)	1.683 (df = 486)	2.143 (df = 497)	2.149 (df = 485)	2.136 (df = 493)	2.149 (df = 493)	2.154 (df = 486)
F Statistic	1.260 (df = 4; 497)	3.646*** (df = 5; 485)	2.386** (df = 5; 493)	1.078 (df = 5; 493)	1.520 (df = 5; 486)	1.655 (df = 4; 497)	2.834** (df = 5; 485)	2.640** (df = 5; 493)	1.448 (df = 5; 493)	1.424 (df = 5; 486)
Note:										*p<0.1; **p<0.05; ***p<0.01

Table 56: General and other adaptation actions with respect to disaster deaths, controls, and interaction terms.

	Dependent variable:									
	General actions					Other actions				
	(1)	(2)	(3)	(4)	(5)	(1)	(2)	(3)	(4)	(5)
deaths	-0.017	-0.218	-0.167	-0.269	-0.867	-0.055	-0.301	-0.146	-0.387	-1.808
	(0.082)	(0.194)	(0.132)	(0.185)	(0.867)	(0.109)	(0.262)	(0.174)	(0.245)	(1.142)
time.lag	-0.047	-0.094	-0.044	-0.038	-0.064	-0.074	-0.106	-0.080	-0.065	-0.083
	(0.098)	(0.098)	(0.099)	(0.098)	(0.099)	(0.129)	(0.133)	(0.131)	(0.130)	(0.131)
city.pop	0.062*	0.069**	0.058**	0.055**	0.072**	0.051	0.072**	0.060*	0.057	0.076**
	(0.032)	(0.027)	(0.027)	(0.027)	(0.029)	(0.042)	(0.036)	(0.035)	(0.035)	(0.038)
deaths*city.pop	-0.013					-0.002				
	(0.021)					(0.027)				
political stability		0.096***					0.092**			
		(0.034)					(0.046)			
deaths*political stability		0.030					0.039			
		(0.027)					(0.036)			
meritocracy			0.133					0.211		
			(0.097)					(0.128)		
deaths*meritocracy			0.105					0.082		
			(0.079)					(0.104)		
stakeholder diversity				-0.058					-0.026	
				(0.090)					(0.119)	
deaths*stakeholder diversity				0.153					0.221	
				(0.115)					(0.151)	
local gov. power					0.064					0.004
					(0.058)					(0.077)
deaths*local gov. power					0.086					0.184
					(0.091)					(0.120)
Constant	1.119***	0.682*	0.954***	1.189***	0.599	1.551***	1.118**	1.298***	1.576***	1.540*
	(0.339)	(0.370)	(0.350)	(0.352)	(0.621)	(0.448)	(0.499)	(0.462)	(0.464)	(0.818)
Observations	505	495	503	503	498	505	495	503	503	498
R^2	0.009	0.036	0.022	0.012	0.016	0.006	0.022	0.017	0.010	0.011
Adjusted R^2	0.001	0.026	0.012	0.002	0.006	-0.002	0.012	0.007	-0.0002	0.001
Residual Std. Error	1.615 (df = 500)	1.582 (df = 489)	1.609 (df = 497)	1.617 (df = 497)	1.620 (df = 492)	2.133 (df = 500)	2.135 (df = 489)	2.126 (df = 497)	2.134 (df = 497)	2.135 (df = 492)
F Statistic	1.181 (df = 4; 500)	3.677*** (df = 5; 489)	2.232** (df = 5; 497)	1.215 (df = 5; 497)	1.594 (df = 5; 492)	0.694 (df = 4; 500)	2.225* (df = 5; 489)	1.715 (df = 5; 497)	0.985 (df = 5; 497)	1.113 (df = 5; 492)
Note:										*p<0.1; **p<0.05; ***p<0.01

Supplementary References

1. Coppedge, M. *et al.* V-Dem Dataset v11.1. *SSRN Electron. J.* (2021) doi:10.2139/ssrn.3831905.
2. Teorell, J. *et al.* The Quality of Government OECD Dataset, version Jan21. *SSRN Electron. J.* (2021) doi:10.2139/ssrn.3786290.
3. African Development Bank. *Poverty and Climate Change: Reducing the Vulerability of the Poor through Adaptation*. (2003).
4. Adger, W. *et al.* Assessment of adaptation practices, options, constraints and capacity. (2007).
5. Brooks, N., Adger, W. N. & Kelly, P. M. The determinants of vulnerability and adaptive capacity at the national level and the implications for adaptation. *Glob. Environ. Chang.* **15**, 151–163 (2005).
6. Gupta, J. *et al.* The Adaptive Capacity Wheel: A method to assess the inherent characteristics of institutions to enable the adaptive capacity of society. *Environ. Sci. Policy* **13**, 459–471 (2010).
7. Smit, B. & Pilifosova, O. From adaptation to adaptive capacity and vulnerability reduction. in *Climate Change, Adaptive Capacity and Development* 9–28 (Imperial College Press, 2003). doi:10.1142/9781860945816_0002.
8. Yohe, G. & Tol, R. S. J. Indicators for social and economic coping capacity - Moving toward a working definition of adaptive capacity. *Glob. Environ. Chang.* **12**, 25–40 (2002).
9. Haddad, B. M. Ranking the adaptive capacity of nations to climate change when socio-political goals are explicit. *Glob. Environ. Chang.* **15**, 165–176 (2005).
10. Williams, C., Fenton, A. & Huq, S. Knowledge and adaptive capacity. *Nat. Clim. Chang.* **5**, 82–83 (2015).
11. Mortreux, C. & Barnett, J. Adaptive capacity: exploring the research frontier. *Wiley Interdisciplinary Reviews: Climate Change* vol. 8 467 (2017).
12. Lemos, M. C. *et al.* Building Adaptive Capacity to Climate Change in Less Developed Countries. in *Climate Science for Serving Society* 437–457 (Springer Netherlands, 2013). doi:10.1007/978-94-007-6692-1_16.
13. Folke, C., Hahn, T., Olsson, P. & Norberg, J. Adaptive Governance of Social-Ecological Systems. *Annu. Rev. Environ. Resour.* **30**, 441–473 (2005).
14. Pahl-Wostl, C. A conceptual framework for analysing adaptive capacity and multi-level learning processes in resource governance regimes. *Glob. Environ. Chang.* **19**, 354–365 (2009).
15. Field, C. B. *Managing the risks of extreme events and disasters to advance climate change adaptation : special report of the Intergovernmental Panel on Climate Change*. (Cambridge University Press, 2012).
16. Vogel, B. & Henstra, D. Studying local climate adaptation: A heuristic research framework for comparative policy analysis. *Glob. Environ. Chang.* **31**, 110–120 (2015).
17. Nightingale, A. J. Power and politics in climate change adaptation efforts: Struggles over authority and recognition in the context of political instability. *Geoforum* **84**, 11–20 (2017).
